# The microbiome stabilizes circadian rhythms in the gut

**DOI:** 10.1073/pnas.2217532120

**Published:** 2023-01-23

**Authors:** Yueliang Zhang, Yongjun Li, Annika F. Barber, Sara B. Noya, Julie A. Williams, Fu Li, Scott G. Daniel, Kyle Bittinger, Jichao Fang, Amita Sehgal

**Affiliations:** ^a^HHMI, University of Pennsylvania, Philadelphia, PA 19104; ^b^Chronobiology and Sleep Institute, Perelman School of Medicine, University of Pennsylvania, Philadelphia, PA 19104; ^c^Institute of Plant Protection, Jiangsu Key Laboratory for Food Quality and Safety-State Key Laboratory Cultivation Base, Ministry of Science and Technology, Jiangsu Academy of Agricultural Sciences, Nanjing 210014, China; ^d^Division of Gastroenterology, Hepatology, and Nutrition, Children’s Hospital of Philadelphia, PA 19104

**Keywords:** circadian rhythms, gut microbiome, timed feeding, transcriptome, circadian phase shifts

## Abstract

The gut microbiome is well known to impact host physiology and health. Given widespread control of physiology by circadian clocks, we asked how the microbiome interacts with circadian rhythms in the *Drosophila* gut. The microbiome did not cycle in flies fed ad libitum, and timed feeding (TF) drove limited cycling only in clockless *per^01^* flies. However, TF and loss of the microbiome influenced the composition of the gut cycling transcriptome, independently and together. Moreover, both interventions increased the amplitude of rhythmic gene expression, with effects of TF at least partly due to changes in histone acetylation. Contrary to expectations, timed feeding rendered animals more sensitive to stress. Analysis of microbiome function in circadian physiology revealed that germ-free flies reset more rapidly with shifts in the light:dark cycle. We propose that the microbiome stabilizes cycling in the host gut to prevent rapid fluctuations with changing environmental conditions.

## Significance

We demonstrate how the microbiome and rhythms of feeding impact circadian rhythms in the gut. Using a *Drosophila* model, we show that restricting feeding to specific times of the day strengthens circadian rhythms of gene expression. However, as compared with ad libitum feeding, time-restricted feeding increases sensitivity of the animals to stressors. This challenges the idea that time-restricted feeding, previously associated with metabolic benefits, promotes fitness. The microbiome, conversely, reduces the strength of circadian cycling in the gut. It also tempers the response of the gut clock to changes in the day:night cycle, thereby allowing circadian rhythms in the gut to remain synchronized with rhythms in the brain. The findings indicate that the microbiome promotes circadian synchrony in the animal.

The gut microbiome plays a critical role in many physiological processes, from metabolism and immunity to even brain function, and thus is considered an important determinant of health and fitness (1–3). Although the microbiome is relatively stable in the long term, short-term fluctuations occur, especially in response to diet (4–6). In addition, the mammalian gut microbiome shows rhythmic variations over the course of a day:night cycle, in terms of its composition as well as its localization within the gut (7–10).

Cycling of the microbiome is dependent on the circadian clock of the host (8–10), and may be mediated through circadian control of food intake. Indeed, rhythms of the microbiome can be restored in clock mutant mice by imposing a feeding rhythm i.e., restricting food availability to a limited interval during the day (8). Such timed feeding (TF) paradigms enhance circadian cycling and have become a popular mode of intermittent fasting (IF) because they improve metabolic health under normal conditions and in mice fed a high-fat diet (11–14). Interestingly, rhythms of the microbiome are abrogated by a high-fat diet, but can be partially restored by TF (10), raising the intriguing possibility that a cycling microbiome contributes to health benefits of TF.

Regardless of whether the microbiome mediates beneficial effects of TF, it affects circadian cycling in different tissues (7, 15–21). The best studied tissue in this regard is the liver, where loss of the microbiome was shown to alter circadian cycling (7, 17, 18). The nature of the effects identified in different studies ranges from the altered phase of rhythmic gene expression to suppressed cycling to a reprogramming of the circadian transcriptome such that different genes cycle in germ-free animals. Weger et al found that loss of the microbiome had minimal effect on clock gene expression in multiple tissues, but dampened sex differences in rhythmic gene expression (20). The relevance of microbiome control of host circadian rhythms is not known, but it is clearly important as circadian disruption impacts physiology in part through the microbiome. Specifically, the microbiome from jetlagged humans confers metabolic deficits, reminiscent of jetlag, to germ-free mice (8).

*Drosophila* have a gut microbiome, which is much simpler than its mammalian counterpart, but is also associated with significant impact on physiology (22). Based upon mammalian data, we expected that the fly microbiome would also cycle and could provide a good model to address the physiological relevance of microbiome cycling. In addition, as a TF paradigm improves cardiac health in aged *Drosophila* (13), we asked if the microbiome mediates effects of TF. We report here that in laboratory-housed *Drosophila*, there is minimal cycling of the microbiome, even under TF conditions. Surprisingly, TF actually compromised responses of the flies to stressors. However, the microbiome has an important circadian role in that it modulates the cycling of clock genes, and tempers responses to shifts in environmental cycles. We propose that it provides stability to the host gut and keeps gut rhythms in synchrony with brain rhythms.

## Results

### The Microbiome Shows Little to No Cycling in *Drosophila*.

**Fig. 1.**
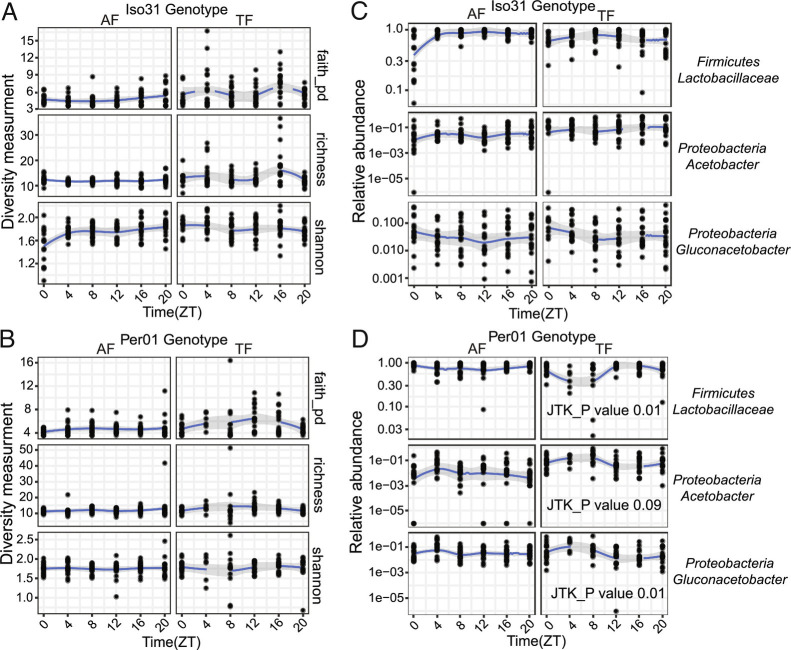
The *Drosophila* intestinal microbiome is largely stable over a daily cycle. (*A* and *B*) Microbiome diversity does not show diurnal oscillations in wild type Iso31 or clock mutant *per^01^* fly guts under ad−lib (AF) or TF conditions. JTK_cycle was used to assess rhythmicity. (*C* and *D*) Specific bacterial species cycle under TF conditions, but only in *per^01^*. JTK_cycle values are shown.

To address circadian regulation of the microbiome, we started by asking if flies fed ad libitum show changes in the microbiome at different times of day. Anticipating that the microbiome would cycle, in which case addressing the clock regulation of it would be important, we assayed wild-type flies (Iso31) as well as flies that lack a key circadian clock gene, *period* (*per*). We collected feces from male and female flies at Zeitgeber Times (ZT) 0, 4, 8, 12, 16, and 20 (ZT0 = lights on and ZT12 = lights off) and subjected these to 16S rRNAseq for analysis of the microbiome (*SI Appendix,* Fig. S1*A* and Dataset S1 *A* and *B*). Using JTK cycle analysis, cycling was not detected for three separate measurements of alpha diversity of the microbiome (Faith's phylogenetic diversity, Shannon diversity, and richness) in either genotype with ad libitum feeding. Similarly, relative abundances of specific species that comprise most of the fly microbiome did not show changes over the course of a day:night cycle (Fig. 1 *A*–*D*, AF condition, which reflects microbiome-containing flies fed ad libitum).

Although flies typically display a circadian rhythm of feeding, we speculated that this might not be robust enough to drive rhythms of the microbiome, so we subjected them to a rhythmic feeding paradigm, such that food was only available from ZT0 to ZT10 (TF condition, i.e., flies carrying a microbiome subjected to TF, in Fig. 1). Similar analysis as above revealed no circadian fluctuation in alpha diversity or taxa abundance in wild-type flies (Fig. 1 *A*–*C*, TF condition). On the other hand, *Lactobacillaceae* and *Gluconacetobacter* both showed periodicity in *per^01^* flies with TF, suggesting that host clocks interfere with the effects of TF on the microbiome (Fig. 1*D*). Aside from circadian rhythms, the overall relative abundance of *Lactobacillaceae* and *Acetobacter* was lower in *per^01^* flies compared with wild type, regardless of feeding conditions (*SI Appendix,* Fig. S1*B*).

### The Microbiome and Rhythmic Feeding Alter the Circadian Transcriptome, Independently and Together.

**Fig. 2.**
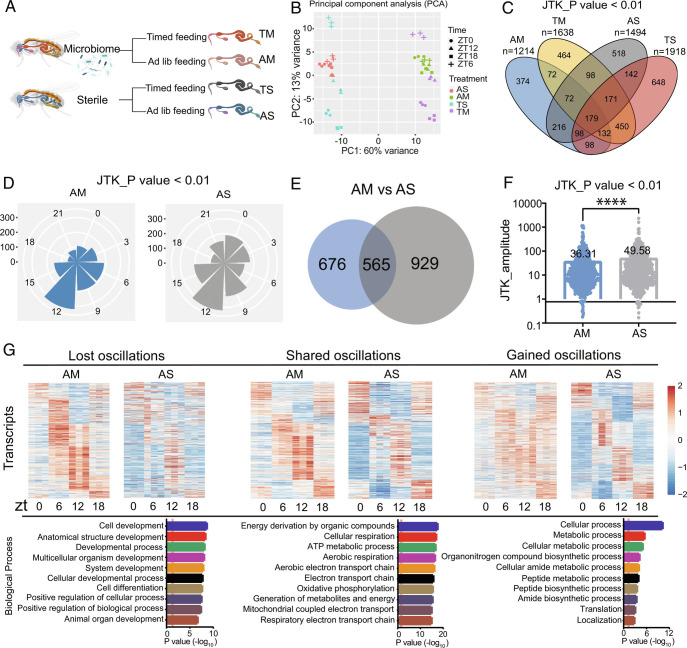
Transcript cycling is enhanced in guts of germ-free flies. (*A*) Schematic showing the experimental protocol for RNA-seq analysis of germ-free (sterile) (S) and microbiome-containing flies (M) under ad lib and TF conditions respectively (AS and TS, AM and TM). Three biological replicates were assayed for each condition. (*B*) Principal component analysis (PCA) of transcript abundance at each timepoint in a 12:12 LD cycle for AM, TM, AS, and TS fly guts. (*C*) Venn diagram illustrates the number of overlapping oscillating transcripts for AM, TM, AS, and TS. JTK_cycle value of *P* < 0.01 was used as the cutoff for cycling. (*D*) Polar histogram plots of the peak phase for oscillating transcripts under AM and AS conditions, using a JTK_cycle value of *P* < 0.01. (*E*) Venn diagram illustrates the number of oscillating transcripts that overlap between AM and AS, using a JTK_cycle cutoff of *P* < 0.01. (*F*) A comparison of amplitudes for cycling transcripts that overlap in AM and AS. Data are mean ± SEM, ****P < 0.0001 shown by Student’s *t* test. (*G*) Phase-sorted heatmaps showing transcripts whose oscillations are lost, shared, or gained in AS flies relative to AM flies. GO biological process enrichment analysis of oscillating transcripts is shown at the *Bottom*.

Although the microbiome was not found to cycle in flies, even with TF, there was reason to believe, from studies in mammals, that it would impact host cycling. To determine if the microbiome, with and without TF, regulates rhythmic gene expression, we depleted female flies, chosen because of the larger amount of material available from them, of their gut microbiome (*SI Appendix,* Fig. S2*A*) and compared circadian gene expression, via RNAseq, in the guts of these animals with that of controls that contained microbiomes. Each condition (sterile or microbiome-containing) was additionally tested under two different feeding paradigms, ad lib and TF, to yield four groups—ad lib sterile (AS), TF sterile (TS), ad lib microbiome (AM), and TF microbiome (TM) (Fig. 2*A*) (Dataset S1 *C*–*F *).

Principal component analysis (PCA) of the top genes expressed in sterile (AS and TS) and microbiome-containing (AM and TM) flies showed a clear separation between the four groups assayed, and indicated an effect of time-of-day, in particular in the groups maintained on TF (Fig. 2*B*). Thus, the circadian transcriptome was distinct for each of the tested conditions. Comparison of these differentially expressed genes also revealed that transcripts enriched in sterile flies are more likely to be expressed rhythmically with TF (clusters C and D in *SI Appendix,* Fig. S2*B*). Nevertheless, several genes were unique cyclers in microbiome-containing flies. Regardless of the statistical cutoff for cycling, the pattern of overlap across the four groups was similar, with some genes expressed cyclically in only one group and some in multiple (Fig. 2*C* and *SI Appendix,* Fig. S2 *C* and *D*). Results with a less stringent cutoff for cycling are shown in *SI Appendix,* Fig. S2 *E*–*H* and *J*–*M*.

The presence of a microbiome, surprisingly, dampened circadian cycling of gut transcripts, although it did not change the overall distribution of phases for the cycling transcriptome (Fig. 2*D*). Enhanced cycling in sterile flies consisted of an increased number of genes expressed rhythmically and also increased amplitude of cycling (Fig. 2 *E* and *F*), and was evident in comparisons of AM versus AS and TM versus TS. Genes that gained cycling in sterile flies, under ad lib conditions, tended to encode metabolic proteins, in particular those involved in amino acid and peptide metabolism (Fig. 2*G*). However, less cycling was observed for genes involved in development and differentiation. Transcripts encoding proteins involved in energy metabolism cycled in sterile and microbiome-containing flies (Fig. 2*G*). Similar results were obtained with less stringent cutoffs for cycling (*SI Appendix,* Fig. S3*A*).

**Fig. 3.**
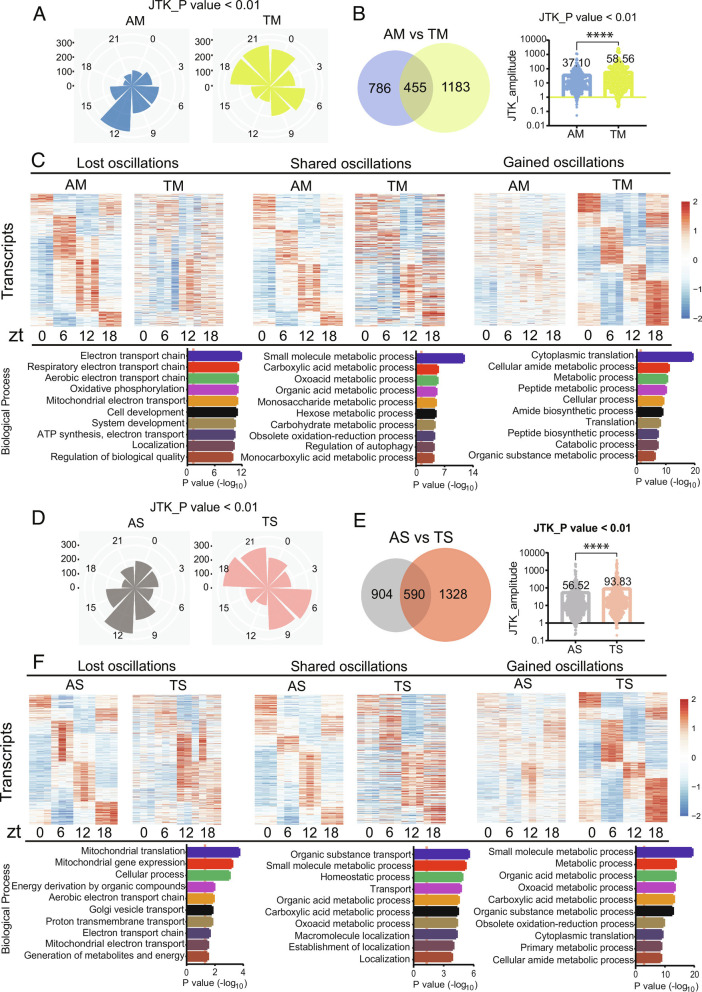
TF changes the phase and amplitude of cycling gut transcripts in germ-free and microbiome-containing flies. (*A*) Polar histogram plots of the peak phase for oscillating transcripts under AM and TM conditions. *P* < 0.01 by JTK_cycle was used as the cutoff for cycling. (*B*) Comparison of amplitudes for transcripts that overlap cycle in AM and TM. Data are mean ± SEM, ****P < 0.0001 shown by Student’s *t* test. (*C*) Phase-sorted heatmaps showing transcripts whose oscillations are lost, shared or gained in TM flies versus AM flies. GO biological process enrichment analysis of oscillating transcripts is shown at the *Bottom*. (*D*) Polar histogram plots of the peak phase for transcripts that oscillate in AS and TS conditions. (*E*) Comparison of amplitudes for transcripts that overlap cycle in AS and TS. Data are mean ± SEM, ****P < 0.0001 shown by Student’s *t* test. (*F*) Phase-sorted heatmaps for transcripts whose oscillations are lost, shared, or gained in TS flies versus AS flies. GO biological process enrichment analysis of oscillating transcripts is shown at the *Bottom*.

TF affected the distribution of phases in the rhythmic transcriptome, in addition to increasing the number of cycling transcripts and the overall amplitude of cycling (Fig. 3 *A*–*F*). This was evident in flies containing a microbiome (Fig. 3 *A*–*C*) and in sterile flies (Fig. 3 *D*–*F*). The change in phase was unexpected as the protocol was designed to match the endogenous rhythm (23). Given that feeding rhythms can vary a bit from strain to strain (24, 25), we assayed the feeding patterns of iso31 flies used here. We detected a peak in the morning, which is consistent with previous reports (23, 26) and suggests that phase differences between AF and TF are not due to altered phase of feeding (*SI Appendix,* Fig. S4*A*). We saw no differences between sterile flies and those that carried a microbiome, indicating that transcriptomic differences under these conditions are also not due to altered feeding (*SI Appendix,* Fig. S4*B*).

Transcripts that gained cycling with TF, or that cycled under both ad lib and TF conditions, encoded metabolic proteins. Interestingly, transcripts involved in mitochondrial/energy metabolism tended to lose cycling under TF conditions (Fig. 3 *C* and *F*). Heat maps using a less stringent cutoff for cycling are shown in (*SI Appendix,* Fig. S3 *B* and *C*).

Consistent with the lack of an effect of the microbiome on the distribution of phase, comparison of sterile flies with microbiome-containing flies under TF conditions did not reveal any differences in phase although more genes cycled in the sterile flies and with higher amplitude (*SI Appendix*, Fig. S5 *A* and *B*). In general, with TF, sterile flies gained cycling of metabolic genes and lost cycling of transcripts implicated in translation and transport (*SI Appendix,* Figs. S3*D* and S5*C*).

To address the overlap between the effects of the microbiome and TF, we asked if the genes that gain cycling in TM flies, relative to AM, also gain cycling in sterile conditions. Of 1,183 genes that show enhanced cycling in TM, 269 displayed enhanced cycling in sterile flies, suggesting partial but not complete overlap (Dataset S1*G*). Given some independent effects of a TF paradigm and the loss of a microbiome, a combination of these two regimens (sterile flies maintained on TF) resulted in maximal cycling. This was reflected in a very high number of cycling genes, and also higher amplitude cycling of genes that cycled across all conditions (*SI Appendix,* Figs. S2*I*, S3*E*, and S5 *D* and *E*). Notable in these shared cycling transcripts were those encoding proteins of small molecule and carboxylic acid metabolism. Changes in cycling brought about by loss of the microbiome or by TF were independent of changes in average expression levels of the affected genes (*SI Appendix,* Fig. S6 *A*–*D*), which is consistent with the findings of Thaiss et al. (7).

### Cycling of Metabolic Transcripts Is Enhanced by TF and Lack of a Microbiome.

As both loss of a microbiome and TF predominantly affected metabolic genes, we closely examined the daily expression of a selection of metabolic genes under AM, TM, AS, and TS conditions (Dataset S1*H*). Increased amplitude with TF or with loss of the microbiome resulted either from higher peak levels or lower trough levels (*SI Appendix,* Fig. S7 *A*–*P*). For instance, *Prx2540-2* showed higher peak levels with TF in both sterile and microbiome-containing flies (TS and TM), while increased amplitude of *Sodh1* rhythms resulted largely from a decrease in trough levels. In general, loss of a microbiome tended to increase overall levels, which often resulted in an increase in amplitude e.g., *Ninad* and *Sodh1*.

### TF and the Microbiome Differentially Affect the Cycling of Clock Genes and Other Transcription Factors.

The microbiome and TF also altered the cycling of clock genes, although probably less so than they did clock-controlled genes (*SI Appendix*, Fig. S8 *A*–*F*), and the effect varied from one clock gene to another. Loss of the microbiome increased the amplitude of cycling of *tim, pdp1,* and *Clk*, but not of *cyc* (*SI Appendix*, Fig. S8 *B*, *C*, *E* and *F*). Changes in the amplitude of *per* were not significant with the four time points assayed in the RNAseq experiment, but were evident in qPCR experiments where six time points were assayed (*SI Appendix,* Fig. S9*A*). TF increased the amplitude of cycling of *per, cry,* and *cyc* in sterile flies, but in microbiome-containing flies it only enhanced *cry* cycling (*SI Appendix*, Fig. S8 *A*, *D* and *E*). *Clk* and *tim* showed no effect of TF and, interestingly, *pdp1* cycling showed a decrease in amplitude with TF of sterile flies (*SI Appendix*, Fig. S8*C*).

As changes in clock genes were variable and did not always parallel changes in the transcriptome as a whole, we asked if microbiome and/or TF-induced alterations in cycling genes were mediated by altered cycling of other transcription factors. Many transcription factors cycled in one or more of the conditions assayed—AM, TM, AS, TS—and, as in the case of other transcripts, cycling was more robust in sterile flies and also with TF (*SI Appendix*, Fig. S9 *C*–*F* and Dataset S1 *I*–*L*). Examination of the distribution of phases revealed that the microbiome had little effect on phase distribution, but TF altered phases of transcription factor cycling in sterile and microbiome-containing flies (*SI Appendix,* Fig. S9*G*). This matches what was observed for the transcriptome as a whole (Figs. 2 and 3 and *SI Appendix,* Fig. S9*H*), and is consistent with other reports of changes in transcription-mediating effects of feeding (27, 28). In addition, specific metabolism-relevant transcription factors responded to TF and loss of a microbiome in ways that parallel effects on other transcripts (*SI Appendix,* Fig. S9 *I*–*N*). We suggest that global changes in cycling genes are downstream of changes in specific transcription factors effected by the reported manipulations, in particular TF.

### Histone Acetylation Is Affected by Rhythmic Feeding and Impacts Levels of Cyclically Expressed Genes.

**Fig. 4.**
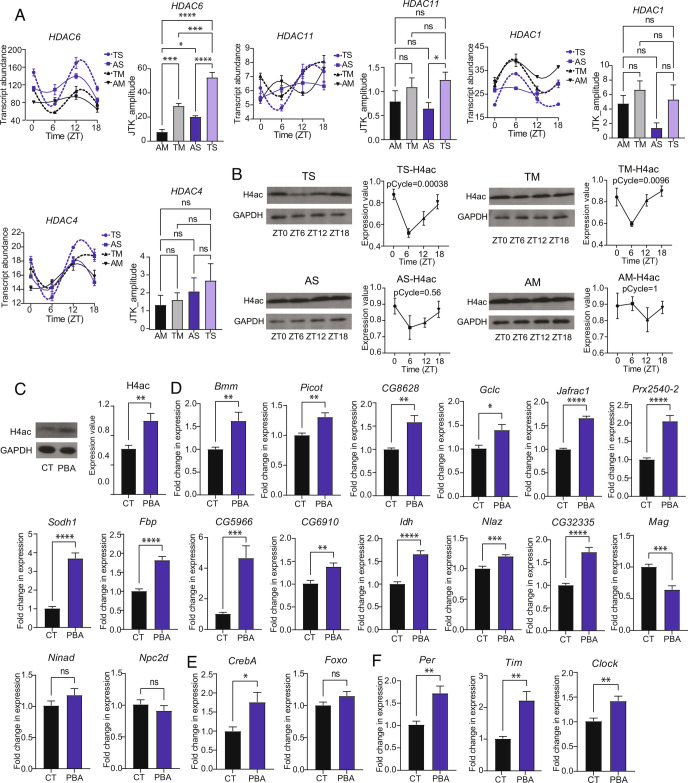
Histone acetylation cycles with TF and regulates the expression of metabolic transcripts. (*A*) Transcript abundance at different times of day and JTK_amplitude of four rhythmically expressed HDAC genes. Expression was assayed under AS, TS, AM, and TM conditions during the RNAseq analysis above. Data are mean ± SEM, *P < 0.05, ***P < 0.001 and ****P < 0.0001 determined by one-way ANOVA and Tukey’s multiple comparison test. (*B*) Western blot shows gut protein levels of H4ac at different timepoints in a 12:12 h LD cycle under AM, TM, AS and TS conditions. Data are mean ± SEM, n = 5 to 7 independent experiments. pCycle indicates presence of a rhythm using JTK cycle analysis. (*C*) Comparison of H4ac protein expression levels 12 h after injection of an HDAC inhibitor (PBA). Data are mean ± SEM, n = 5 biological replicates, **P < 0.01 determined by Student’s *t* test. (*D*) Expression of 16 representative metabolic genes 12 h after injection of PBA. Data are mean ± SEM, *P < 0.05, **P < 0.01, ***P < 0.001 and ****P < 0.001 determined by Student’s *t* test. N = 7 biological replicates. (*E* and *F*) Expression of two representative transcription factors (*CrebA* and *FOXO*) and three clock genes (*per*, *tim* and *clock*) 12 h after injection PBA. Data are mean ± SEM, *P < 0.05 determined by Student’s *t* test. N = 7 biological replicates.

Given the profound effects of the microbiome and TF on host gene cycling, we asked what mechanisms might mediate these effects. Histone acetylation is implicated in the impact of the mouse microbiome on host gene expression (15), so we considered it as a mechanism here. We found that TF enhanced the cycling of several histone deacetylases (HDACs) and also drove cycling of acetylated histone H4 (H4ac) in the guts of sterile flies (Fig. 4 *A* and *B*). TF had less of an effect on flies that contain a microbiome, although it still drove significant cycling (Fig. 4 *A* and *B*). Acetylated H4 was not detectably different between sterile flies and microbiome-containing flies (Fig. 4*B*), so we focused on effects of TF.

To determine whether histone acetylation could account for the enhanced cycling seen with TF, we injected ad lib fed iso31 flies with histone deacetylase inhibitors and examined gene expression in fly bodies, focusing on genes that showed stronger cycling with TF. The HDAC inhibitor sodium phenyl-butyrate (PBA) increased expression of H4ac (Fig. 4*C*) and also increased expression of many of the genes expressed cyclically with TF, implicating TF-driven rhythms of histone acetylation in the cycling of these genes (Fig. 4*D*). A different HDAC inhibitor, Valproic acid (VA), did not consistently increase H4ac, but still increased levels of many genes, including the transcription factor *CrebA* and clock genes *per, tim,* and *Clk* (*SI Appendix,* Fig. S10 *A*–*C*), perhaps through effects on other histones. Some genes were preferentially affected by one HDAC inhibitor, PBA or VA, and not the other (*SI Appendix,* Fig. S10*D*). Given the effects of TF on histone acetylation, together with enhanced expression of genes targeted by TF with HDAC inhibitors, we propose that histone acetylation is a major mechanism mediating effects of TF on the circadian transcriptome.

### TF Does Not Increase Resistance of Flies to Stress.

We next sought to determine the functional impact of the microbiome and TF on physiology, asking also whether beneficial effects of these were linked. For instance, TF paradigms promote metabolic health, and so we wondered if the microbiome was required for the health benefits. To establish a paradigm to visualize beneficial effects of TF, we focused on responses to stress, as these constitute a good measure of fitness. Thus, we exposed 5 to 7-d-old flies to ad lib feeding conditions or TF for 4 or 21 d (*SI Appendix,* Fig. S11*A*) and then treated them with different stressors—starvation, injection with bacteria, heat shock—and assayed survival. The 21-d protocol was based on a previous study where this duration was required for beneficial effects of TF (29). Surprisingly, while the 4-d TF had no effect on the response to starvation, TF for 21 d actually decreased survival (*SI Appendix,* Fig. S11 *B* and *C*). In our initial experiments, TF consisted of a 14-h fast (food availability from ZT0 to 10, as above), but we found that decreasing the duration of the fast to 10 h did not change the outcome of the experiment. As with the 14-h fast, the 10-h fast actually decreased survival in the 21-d protocol (*SI Appendix,* Fig. S11*B*).

To determine whether the TF paradigm affected the health of the flies in obvious ways, especially after 21 d, we first measured the weight of female flies after 4 and 21 d of each of the three TF paradigms used. While the weight of flies maintained on TF was a little lower than that of ad lib fed flies, the difference was the same at 4 and 21 d, indicating that changes in weight did not account for the different responses at these two time points (*SI Appendix,* Fig. S12 *A* and *B*). We also measured locomotor activity of flies subjected to the different feeding conditions, using multibeam *Drosophila* Activity Monitors (DAM) that allow high-resolution monitoring of activity. The activity of females, but not males, was higher in some TF flies than those fed ad lib, but it was not always with the same TF paradigm nor was it associated with the duration of the fast. Also, the data for this parameter were similar with 4- and 10 d of TF (*SI Appendix,* Fig. S12 *C* and *D*). Thus, decreased survival with TF is not due to obvious impairments in health/function and likely results from changes in endogenous metabolism.

**Fig. 5.**
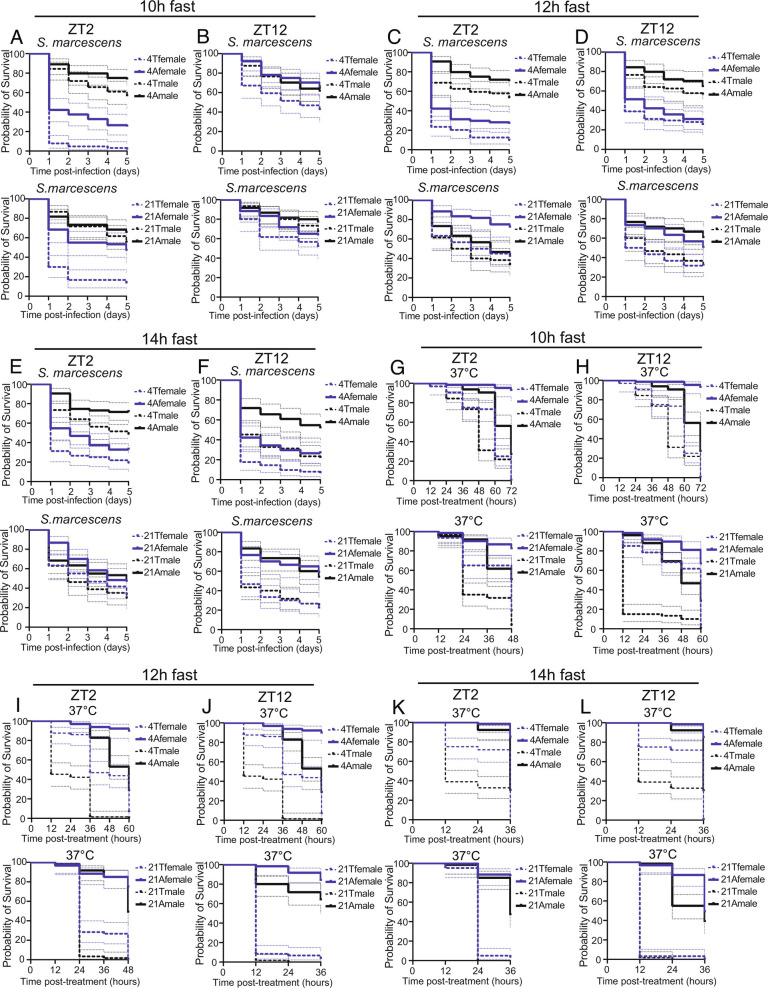
Flies kept on a TF paradigm are more sensitive to bacterial infection and heat shock stress. (*A*–*F*) Female and male flies under ad lib or TF treatments, as indicated, were intrathoracically injected with *S.marcescens* then the survival was measured daily. (*A*) Flies were infected at ZT2 after a 10 h fast each day for 4 or 21 d. (TF (T) versus AF (A), 4 d female *P* value <0.001, 4-d male *P* < 0.05, 21-d female *P* < 0.001, 21-d male *P* = 0.36). (*B*) Flies were infected at ZT12 after a 10 h fast each day for 4 or 21 d (TF versus AF, 4-d female *P* < 0.004, 4-d male *P* = 0.7, 21-d female *P* = 0.15, 21-d male *P* = 0.27). (*C*) Flies were infected at ZT2 after a 12-h fast each day for 4 or 21 d (TF versus AF, 4-d female *P* < 0.02, 4-d male *P* < 0.04, 21-d female *P* < 0.0005, 21-d male *P* = 0.18). (*D*) Flies were infected at ZT12 after a 12-h fast each day for 4 or 21 d (TF versus AF, 4-d female *P* = 0.42, 4-d male *P* = 0.30, 21-d female *P* < 0.016, 21-d male *P* < 0.0014). (*E*) Flies were infected at ZT2 after a 14-h fast each day for 4 or 21 d (TF versus AF, 4-d female *P* < 0.05, 4-d male *P* < 0.008, 21-d female *P* = 0.083, 21-d male *P* = 0.061). (*F*) Flies were infected at ZT12 after a 14-h fast each day for 4 or 21 d (TF versus AF, 4-d female *P* < 0.0013, 4-d male *P* < 0.0002, 21-d female *P* < 0.0001, 21-d male *P* < 0.0001). (*G*–*L*) Female and male flies under ad lib or TF treatments, as indicated, were exposed to 37 °C then the survival was measured every 12-h. For all conditions, TF reduced survival as compared with AF, *P* < 0.0001). (*G*) Flies were exposed to 37 °C at ZT2 after a 10-h fast each day for 4 or 21 d. (*H*) Flies were exposed to 37 °C at ZT12 after a 10-h fast each day for 4 or 21 d. (*I*) Flies were exposed to 37 °C at ZT2 after a 12 -h fast each day for 4 or 21 d. (*J*) Flies were exposed to 37 °C at ZT12 after a 12-h fast each day for 4 or 21 d. (*K*) Flies were exposed to 37 °C at ZT2 after a 14-h fast each day for 4 or 21 d. (*L*) Flies were exposed to 37 °C at ZT12 after a 14-h fast each day for 4 or 21 d.

TF also decreased survival upon bacterial infection or heat shock in both sexes. In these experiments, we tested effects of 10-h, 12-h, or 14-h fasts, in each case initiating feeding at ZT0. However, in general, fasting was deleterious to the survival of flies in response to infection or heat shock (Fig. 5 *A*–*L*). We infer that while TF promotes metabolic health, it may not be optimal for responses to stress.

### Sterile Flies Lacking a Microbiome Reset More Rapidly with Shifts in the Light:Dark Cycle.

**Fig. 6.**
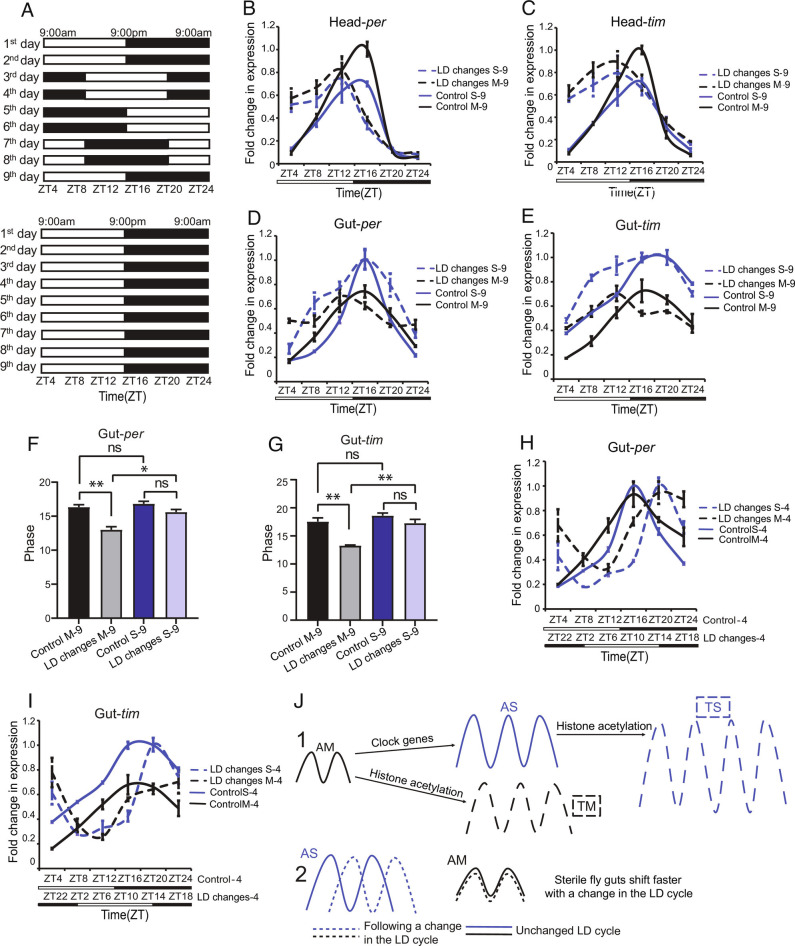
Gut clocks show slower adaptation to changes in the light:dark cycle in microbiome-containing flies. (*A*) Schematic showing the experimental protocol used to produce changes in the light:dark cycle and the sampling time-points for female guts or heads. (*B* and *C*) Expression of head *per* and *tim* in AM and AS lines at different timepoints on the ninth day. mRNA levels were measured by qPCR and normalized to RP49. Data are mean ± SEM (n = 3). (Control AM: black line; light:dark changes AM: dashed black line; Control AS: blue line; light:dark changes AS: dashed blue line). (*D* and *E*) Expression of gut *per* and *tim* mRNA in AM and AS lines at different timepoints on the ninth day. mRNA levels were measured by qPCR and normalized to the RP49. Data are mean ± SEM (n = 3). (*F* and *G*) Comparison of relative peak time values of *per* and *tim* RNA in AM and AS guts on the ninth day, i.e., following LD changes. Peak times were based on meta2d analysis. Data are mean ± SEM, *P < 0.05, **P < 0.01, and ***P < 0.001 determined by Student’s *t* test. (*H* and *I*) Expression of gut *per* and *tim* in AM and AS lines at different times of day on the fourth day. mRNA levels were measured by qPCR are normalized to RP49, data are mean ± SEM (n = 4), (Control AM: black line; light:dark changes AM: dashed black line; Control AS: blue line; light:dark changes AS: dashed blue line). (*J*) Model showing effects of the microbiome and TF on molecular rhythms in the gut. RNA rhythms are strengthened by TF or loss of a microbiome (AS). When these two manipulations are coupled, rhythms become even stronger (TS). *Bottom* shows that sterile flies shift more rapidly with changes in the LD cycle.

As the fitness paradigm we used above did not show benefits of TF, we could not use it to determine if the microbiome mediates beneficial effects of TF. Instead, we considered other ways to assess the functional impact of the microbiome. Although, the microbiome is well studied in terms of its impact on metabolism and immunity (1–3), little is known about its physiological relevance to the circadian system, which is also critical for health. Flies with a microbiome show reduced amplitude circadian cycling, relative to sterile flies, raising the question of whether the dampened cycling is beneficial to the host. To determine if resetting of the gut clock by different environmental cycles is affected by the microbiome, we subjected sterile and microbiome-containing flies to a circadian phase shift paradigm where every 2 d they received a 6-h delay in the light: dark (LD) cycle (Fig. 6*A*). On the ninth day, they ended up back on the original LD cycle, and during this day we examined circadian expression of the genes *per* and *tim* in heads and guts and compared it with that of controls that had remained in the original LD cycle throughout (Fig. 6 *B*–*E*).

On day 9, *per* and *tim* expression in heads of experimental flies was different from that of unshifted controls, indicating that it had not shifted back to the original cycle (Fig. 6 *B* and *C*, *SI Appendix,* Fig. S13*A*). We noted that, although the presence of the microbiome reduces amplitude of circadian gene expression in the gut, it appeared to increase cycling of *per* and *tim* in heads. In guts of microbiome-containing flies subjected to light:dark changes, *per* and *tim* expression was similar to that in heads, i.e., it was different from unshifted controls and not resynchronized to the LD cycle on the ninth day (Fig. 6 *D* and *E*). Surprisingly, *per* and *tim* expression in sterile flies subjected to LD changes showed a profile indistinguishable from that of controls, indicating that these flies adapted very quickly to the LD cycle on Day 9 (Fig. 6 *F* and *G*). *tim* expression in these flies did not shift as quickly, but even so, sterile flies showed a significant shift to match the phase of unshifted controls.

As the phase of the sterile flies at the end of the experiment matched that of controls that remained in the same LD cycle throughout, we sought to confirm that the sterile flies had shifted in response to the light:dark changes earlier in the experiment. We first examined day 3 of the experiment, which represents the first day of a new LD cycle and thus is roughly equivalent to day 9, although on day 3, the shift to the new LD involves an 18-h dark period and the shift on day 9 introduces an 18-h light period. On day 3, *per* and *tim* showed transitional phases, and there was no obvious difference between sterile and microbiome flies (*SI Appendix,* Fig. S13*B*), suggesting that the microbiome influences responses to light and so has less of an effect in extended darkness. We then examined *per* and *tim* expression in guts on the second day after the first move to a new LD cycle, i.e., day 4 of the experiment. In this case, *per* expression had shifted to the new regime in sterile and microbiome-containing flies. *tim* expression showed a pronounced shift in sterile flies, but not in microbiome-containing flies, where it still showed a transitional phase (Fig. 6 *H* and *I*). We surmise that in the gut, the expression of *tim* RNA shifts slower than *per* RNA, but both shift faster in sterile flies than in flies that have a microbiome. Thus, the presence of a microbiome stabilizes the gut clock in the event of environmental changes.

## Discussion

We report here that the *Drosophila* microbiome does not cycle, but it regulates the gut circadian transcriptome and prevents rapid fluctuations in response to environmental cycles (Fig. 6*J*).

Given reports of a cycling microbiome in the mammalian gut, it is surprising that the *Drosophila* analog does not cycle (7–10). The reason for this is unclear. The composition may not cycle because the fly biome has relatively low diversity, comprised largely of *Lactobacilllus* and *Acetobacter* species that may be required at all times (30). On the other hand, the abundance of bacteria in the fly gut is driven almost entirely by feeding (31), so it is possible that flies eat throughout the day. Indeed, while ad lib flies display a circadian rhythm of food intake, the rhythm is not very robust and feeding is also evident during off-peak times (23, 25, 26) (*SI Appendix*, Fig. S4). If food is the major stimulus, one would expect a TF paradigm to drive cycling, but even under these conditions limited cycling was observed only in clockless flies. It is possible that the internal clock inhibits cycling of the microbiome, perhaps because a cycling microbiome is not beneficial to flies.

The TF protocol failed to drive cycling of the microbiome, and did not provide protection to the host in response to different stressors. We asked if TF increases resistance to stress if imposed for >20 d rather than 4 d; in this case, flies are older when assayed for stress responses, and so they are chronologically closer to the aged flies previously reported to show enhanced cardiovascular health upon TF (13). However, we still saw no effect. Thus, TF promotes metabolic health, but stress responses may require substantial nutrient sources that could be compromised by limited feeding. This is supported by studies showing that caloric restriction, which increases lifespan across species, increases susceptibility to intact pathogens, even while enhancing immune function (32, 33). We note too that the standard TF paradigm used here and previously (13) did not extend *Drosophila* lifespan. A different IF protocol needed to be used (34), suggesting, additionally, that TF protocols need to be tweaked for lifespan extension in *Drosophila*.

Each of the two manipulations we studied – TF and loss of the microbiome – did, however, have significant impact on the cycling transcriptome in the host gut. As reported previously for mammals, the composition of the cycling transcriptome changed in flies lacking a microbiome (7, 20). Transcripts encoding metabolic proteins exhibited increased cycling in sterile flies, perhaps to compensate for loss of metabolic properties that would normally be conferred by the microbiome. On the other hand, less cycling was observed for genes involved in development and differentiation, which may be required for the gut to accommodate the microbiome. Particularly striking was the loss of cycling of transcripts involved in oxidative phosphorylation and energy metabolism under TF conditions. We speculate that TF directly impacts the activity of cellular processes that generate energy (e.g., mitochondrial function), and so cyclic expression of the genes encoding the relevant proteins is not required.

We report also that the amplitude of cycling was enhanced by TF or loss of the microbiome. TF not only enhanced cycling and changed the composition of the transcriptome, it also shifted the phase of gene expression. Genes whose expression cycles only under TF likely reflect those that respond to food intake but are not driven by the clock. Clock genes showed varied responses to TF and to loss of the microbiome, raising the question of the extent to which they contribute to the cyclic transcriptome. Interestingly, analysis of cycling transcription factors yielded results that closely match those seen for the entire transcriptome, suggesting that global changes in cycling are a consequence of altered activity of upstream transcription factors. This lines up well with previous reports that examined effects of TF (27, 28). Whether cycling transcription factor activity depends on the clock remains to be determined, although much of it likely is. On the other hand, some of the histone acetylation we report here (see below) could bypass the clock.

Increased amplitude of cycling with TF appears to be due to increased histone acetylation. We find that TF drives robust rhythms of histone acetylation, especially in sterile flies, and many of the genes affected by TF are responsive to histone acetylation. The latter is demonstrated by increased expression of these genes upon treatment with HDAC inhibitors, although we acknowledge that one of the inhibitors used here (PBA) can also have other effects (35). Also, not all genes that show enhanced cycling with TF are responsive to HDAC inhibitors (e.g. see Fig. 4*C* and *SI Appendix*, Fig. S10), indicating that other mechanisms are also involved.

At first glance, it appears that our microbiome data are different from previous mammalian studies in terms of showing that the biome dampens host cycling. However, the only real difference is from the work of Leone et al. who found that loss of the microbiome suppresses host cycling (17). Although Gachon et al. focused more on sex differences, their data show better cycling in axenic animals (20). Montagner et al. reported altered cycling in animals that lacked a microbiome and, again, examination of their data suggests enhanced amplitude (18). Finally, Thaiss and Gachon showed changes in the composition of the circadian transcriptome, which is consistent with our data (7, 20).

The fact that the microbiome weakens clock gene cycling runs contrary to the thinking that cycling benefits the host and so should be enhanced by a fitness-conferring microbiome. However, lower amplitude cycling of the host gut transcriptome could be beneficial as it might allow it to be more easily synchronized with other body tissues. For instance, we find here that gut cycling is in synchrony with cycling in the head when microbiome-containing flies are moved from one light:dark cycle to another. On the other hand, the clock in the microbiome-containing gut is less sensitive to changes in the light:dark cycle (note that the fly gut may be directly light-responsive), which prevents random fluctuations in circadian function. Indeed, reduced responsiveness to light could also account for the weaker cycling in these flies. In sterile flies, clock gene cycling in the gut is very sensitive to environmental light:dark changes and so it shifts even when cycling in the head does not. Overall, our data suggest that a robust microbiome stabilizes circadian cycling in the gut to promote synchrony within the organism and resist rapid changes in the environment. These findings have important implications for common situations in the modern world.

## Materials and Methods

### Generation and Maintenance of Fly Lines.

The *w^118^ iso^31^, per^01^* fly lines present in the study were maintained on standard cornmeal/molasses medium at 25 °C under 12:12 LD conditions unless otherwise specified. For 16S sequencing of gut microbiomes, we first treated flies with 1 mM kanamycin (11815024, ThermoFisher) to remove *Wohlbachia*, as this would otherwise swamp other bacterial signals in the sequencing. Afterward, the flies were repopulated with a Wolbachia-free microbiome containing *Lactobacillus* and *Acetobacter bacteria* from medium previously occupied by other flies. Generation of sterile flies and an account of the TF protocol is provided in the (**SI Appendix*, *SI Methods**).

### Sample Collection and 16S Sequencing.

5 to 7–d old female and male flies were separately transferred into fresh sterile medium vials every 4 h. At least 35 flies were maintained per vial and allowed to defecate at 25 °C under 12:12 LD conditions coupled with AF or TF treatment method as above. Processing of fecal samples for 16S sequencing is described in the (**SI Appendix*, *SI Methods**).

### Immunity and Antistress Test.

For heat shock and infection stress tests, 4-d-old flies were maintained in 12:12 light:dark cycles and fasted for 10-h, 12-h, or 14-h each day and then tested after either 4 d or 21 d. Details of the test can be found in the *SI Appendix*, *SI Methods*.

### RNA-seq and Data Analysis.

For each RNAseq experiment, total RNA was extracted from at least 30 male guts by using the SV Total RNA Isolation kit (Z3105, Promega). Details of RNA isolation, preparation of libraries and RNAseq analysis can be found in the (*SI Appendix*, *SI Methods*).

### HDAC Inhibitor Administration.

Each of the two histone deacetylases inhibitors, sodium 4-phenylbutyrate (PBA) (HY-15654, MedChenExpress) and VA (PHR1061, Sigma), was dissolved in PBS containing 1% blue food coloring and injected subcutaneously into the female fly body. Injection concentrations for PBA and VA were 30 mM and 10 mM, respectively. PBS containing 1% blue food coloring was used as a control. Histone acetylation and target gene expression were investigated 12 h after injection of either inhibitor, by western blot or qPCR. Mortality of the flies was also monitored throughout the experiment.

### Quantitative Real-Time PCR and Western Blot Analysis.

Details are provided in the **SI Appendix*, *SI Methods**.

### Circadian Phase Shift Assay.

For the circadian phase shift assay, microbiome-containing and sterile female flies were subjected to a 6-h delay in the LD cycle every 2 d, i.e., on the third, fifth, seventh, and ninth days, such that flies returned to the original phase on Day 9. Female fly guts were dissected at ZT4, ZT8, ZT12, ZT16, ZT20, and ZT24 timepoints. Control flies were maintained in the original LD regime (9 am:9 pm) at 25 °C. For each time point, three repeat samples (>30 guts per sample) were collected for microbiome-containing and sterile female flies, respectively. All the samples were subjected to qPCR analysis.

### Statistical Analysis.

Statistical details of experiments can be found in the figure legends. Circadian statistical analysis was performed in R using JTK_CYCLEv3.1. One-way ANOVA with Holm–Sidak post hoc correction tests were performed with GraphPad Prism 9, experimental groups were compared by unpaired *t* test with Welch’s correction. *P < 0.05, **P < 0.01, ***P < 0.001, ****P < 0.0001. Error bars represent ± SEM, unless otherwise stated in the figure legends.

## Supplementary Material

Appendix 01 (PDF)

Dataset S01 (XLSX)

Dataset S02 (XLSX)

## Data Availability

Microbiome data are available at NCBI BioProject with accession number PRJNA923004. Metadata for RNAseq analysis are available at https://dataview.ncbi.nlm.nih.gov/object/PRJNA922929?reviewer=dteltea5m87afl41vn49jrche5. Some study data are included in the article and/or *SI Appendix*.
