# Supplementary material for: The microbiome stabilizes circadian rhythms in the gut
Source: Proc Natl Acad Sci U S A. 2023 Jan 23;120(5):e2217532120. doi: 10.1073/pnas.2217532120 (PMC9945975; doi:10.1073/pnas.2217532120)
Supplement: Supplementary file 1 — Appendix 01 (PDF) [file pnas.2217532120.sapp.pdf]

Supporting Information for

The microbiome stabilizes circadian rhythms in the gut

Yueliang Zhang [a,b,c], Yongjun Li [a,b], Annika F. Barber [a,b], Sara B. Noya [a,b], Julie A Williams [a,b], Fu Li [a,b], Scott G Daniel [d], Kyle Bittinger [d], Jichao Fang [c*], Amita Sehgal [a,b*].

[a]HHMI, University of Pennsylvania, Philadelphia, PA 19104. [b]Chronobiology and Sleep Institute, Perelman School of Medicine, University of Pennsylvania, Philadelphia, PA 19104. [c]Institute of Plant Protection, Jiangsu Key Laboratory for Food Quality and Safety−State Key Laboratory Cultivation Base, Ministry of Science and Technology, Jiangsu Academy of Agricultural Sciences, Nanjing, China, 210014. [d]Division of Gastroenterology, Hepatology, and Nutrition, Children's Hospital of Philadelphia, PA 19104.

* Amita Sehgal and Jichao Fang

Email: amita@pennmedicine.upenn.edu or fangjc@jaas.ac.cn

This PDF file includes:

Supporting text
Figures S1 to S13
Legends for Datasets S1A-M
SI References

Supplement materials and methods
SI Materials and Methods

Generation and maintenance of fly lines

Sterile flies were generated with the Wohlbachia-minus iso31 fly line. Newborn fly embryos within 12 hours were rinsed in 100% ethanol, dechorionated in 10% bleach for 2 minutes, and immediately rinsed three times in sterile PBS. The sterile embryos were fed autoclaved standard sterile molasses-cornmeal-yeast medium containing 1 mM kanamycin, 650 μM ampicillin (61-238-RH, MediaTech), and 650 μM doxycycline (D9891, Sigma-Aldrich). Germ-free fly lines were maintained on sterile medium with three antibiotics for four generations and then maintained on the same medium without antibiotics for the next four generations. Bacterial contamination of flies was monitored by homogenizing larvae and testing for bacterial growth on DeMan, Rogosa and Sharpe (MRS)-agar plates.

For timed feeding, adult flies were provided standard sterile medium in groups of 40 for 3 days. 5-7 day old flies were separated by gender and subjected to the feeding paradigm in the presence of light:dark cycles starting on day 5. Ad libitum (AF) and flies subjected to timed feeding (TF) were shifted to sterile medium vials at zeitgeber time 0 (ZT0), (which corresponded to actual time of 9:00 am) and then were transferred to either a new sterile medium or a 1.1% agar vial at ZT10 (7pm), ZT12 (9pm) or ZT14 (11pm) for 14-h, 12-h or 10-h fast respectively.

Sample collection and 16S sequencing

Feces from the walls of the sterile tube were collected by using sterile cotton swabs at time points ZT0, ZT4, ZT8, ZT12, ZT16 and ZT20. For each strain, iso31 or per^{01} at least 4 replicates were included at all time points for female and male flies respectively. The spin swab with feces was transferred into a reaction tube and the stick was carefully cut with sterile scissors. DNA was extracted by using the Qiagen DNeasy PowerSoil Pro kit and extracted DNA was quantified with the Quant-iT PicoGreen Assay Kit.

For 16S rRNA marker gene sequencing, PCR amplification was performed using primers that targeted the V1-V2 region of the 16S rRNA gene, followed by sequencing using 250 bp paired-end sequencing on an Illumina MiSeq instrument. Sequence data were analyzed using the QIIME2 bioinformatics pipeline [PMID 31341288]. Taxonomic abundances were compared using linear mixed effects models after log transformation. Cyclic patterns in alpha diversity and taxon abundance were detected using the JTK_Cycle method from the MetaCycle R package.

Immunity and anti-stress test

For infection, LB containing bacteria (*S. marcescens*) was diluted in PBS (to OD600=0.02) together with 1% food coloring (Brilliant Blue FCF). The injection control solution contains equivalent amounts of LB medium and 1% blue food coloring in PBS. Bacterial and control solutions were stored on ice until the time of injection, at ZT2 or ZT12. Survival of infected flies was measured daily by counting the number of live flies in each test tube. For the heat shock experiment, flies were

shifted to 37°C after 4 days and 21 days continuously fast and then surveyed every 12-h for survival rate. For the starvation test, AF and TF flies were transferred into 1.1% agar vials at 25°C under 12:12 LD conditions and surveyed every 12-h for survival rate. Each experiment was repeated at least 4 time, with 15-16 flies per repetition.

RNA-seq and data analysis

The quality of RNA isolated with the SV Total RNA Isolation kit (Z3105, Promega) was assessed by Fragment Analyzer High Sensitivity RNA Assay (DNF-471-0500, Agilent Technologies) and quantified by Qubit 2.0 RNA HS assay (Q32855, ThermoFisher). Paramagnetic beads coupled with oligo d(T)25 were combined with total RNA to isolate poly(A)+ transcripts based on the NEBNext® Poly(A) mRNA Magnetic Isolation Module manual (E7490L, NEB). Prior to first strand synthesis, samples were randomly primed (5´ d (N6) 3´ [N=A,C,G,T]) and fragmented based on manufacturer's recommendations. The first strand was synthesized with the Protoscript II Reverse Transcriptase with a longer extension period, approximately 40 minutes at 42 ºC. All remaining steps for library construction were according to the NEBNext® Ultra™ II Non-Directional RNA Library Prep Kit for Illumina® (E7760L, NEB). Final libraries were quantitated by Qubit 2.0 (Q32855, ThermoFisher) and quality was assessed by TapeStation D1000 ScreenTape (5067- 5582, Agilent). Illumina® 8-nt dual-indices were used. Equimolar pooling of libraries was performed based on QC values and sequencing was conducted by Admera on an Illumina® NovaSeq S4 (Illumina, USA) with a read length configuration of 150 PE for 40 M PE reads per sample (20 M in each direction). For RNA-sequence analysis, FASTQ data were mapped to the fly genome (Number) by using Hisat2 (http://daehwankimlab.github.io/hisat2/) and alignment results were assayed by the LiBiNorm tool (https://warwick.ac.uk/fac/sci/lifesci/research/libinorm/) based on the reference genome GENCODE. Raw data count was analyzed by IDEP v0.95 (http://bioinformatics.sdstate.edu/idep/) for differentially expressed genes (DEGs) between different conditions. The threshold value CPM>0.5 and n libraries >3 and regularized log transformation were respectively used to filter out low-expressed genes and transform raw count data for further clustering and PCA analysis, then the TPM data were used for rhythmicity analysis through Nitecap (https://nitecap.org/) and MetaCycle (https://academic.oup.com/bioinformatics/article/32/21/3351/2415176). Cycling transcription factors were identified through (https://flybase.org/reports/FBgg0000745.html). Each condition assayed in the RNAseq experiment was represented by three biological replicates.

Quantitative real-time PCR and Western blot analysis

Total RNA was extracted by using an RNA kit (Z3105, Promega) and cDNAs were synthesized by using a high-capacity cDNA Archive kit (4374966, Applied Biosystems). Assays were run on a ViiA7 Real-Time PCR system (prism 7100, Applied Biosystems) by using a SYBR Green kit (4364346, Applied Biosystems).

Relative gene expression was calculated using the $2^{-\Delta\Delta CT}$ method normalizing to RP49. Primer sequences are listed in Dataset S1M.

For Western blot analysis, female bodies (flies injected with HDAC inhibitors) or male guts from flies subjected to ad libitum or 14-h timed feeding regimes at indicated time points were collected for protein extraction. All the protein samples were isolated by using the lysis buffer (87788, Thermo Scientific) with protease inhibitor (11873580001, Roche). Primary antibodies Rabbit anti- Acetylated Histone H4 (AHP418, Bio-Rad) and Rat anti-GAPDH (10494-1-AP, Proteintech) were used at 1:1000 and 1:1000 dilution, respectively. FIJI ImageJ software was used for analysis.

Fly feeding rhythm, activity and weight assays

The CAFE feeding assay was performed as described previously with small modifications (1). In brief, plastic vials containing agar on the bottom were placed in a custom made glass chamber filled with water that allowed air flow while maintaining high humidity. Glass micropipettes (5ul, catalog no. 53432-706; VWR, West Chester, PA) were filled with 1 cm of mineral oil and liquid medium containing 5% sucrose and 0.05% Brilliant Blue. The experiment was conducted under an 12:12 LD cycle in an incubator at 25℃ and >60% humidity in the chamber. Four male flies (5-7 day old) per vial were trained for 12-h. During the assay, capillaries were measured and replaced every 3-h across one day (ZT1, ZT4, ZT7, ZT10, ZT13, ZT16, ZT19, ZT23). At every time point a vial without flies was used to control for evaporation and the change in volume was subtracted from the experimental readings.

Fly activity was recorded by using an updated multi-beam (MB) version of the *Drosophila* Activity Monitor (DAM) system, in which 15 beams of infrared detectors were used to detect the movement of single fly in a glass locomotor tube. We used the total number of movements (MT) data collected by the MB-DAM system, as it reflects the breaking of two beams, and thereby active locomotion of the fly. The total and mean MT per minute over 10-h after fly loading were calculated by R and used to indicate the fly's physical activity.

An electronic balance (Sartorius CP124S, precision 0.1 mg) was used for weighing fly bodies in groups of 100.

Fig.S1

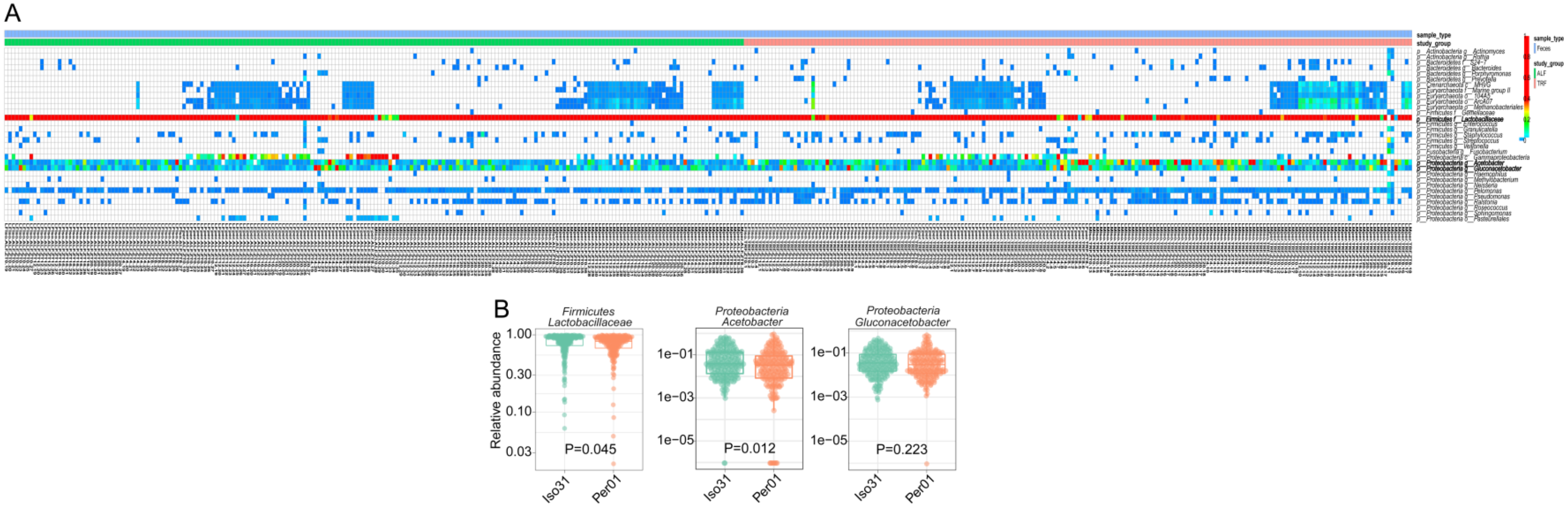

Figure S1. Abundance of three bacterial species in Iso31 and *per*[01] lines

A: Heatmap representation of global bacterial abundance at six timepoints in a 12:12 h LD cycle for Iso31 and *per*[01] flies with ad−lib and timed feeding treatments. **B:** Relative abundance of three gut bacterial species in Iso31 and *per*[01] lines. Data are mean ± SEM, p value determined by student's t test.

Fig.S2

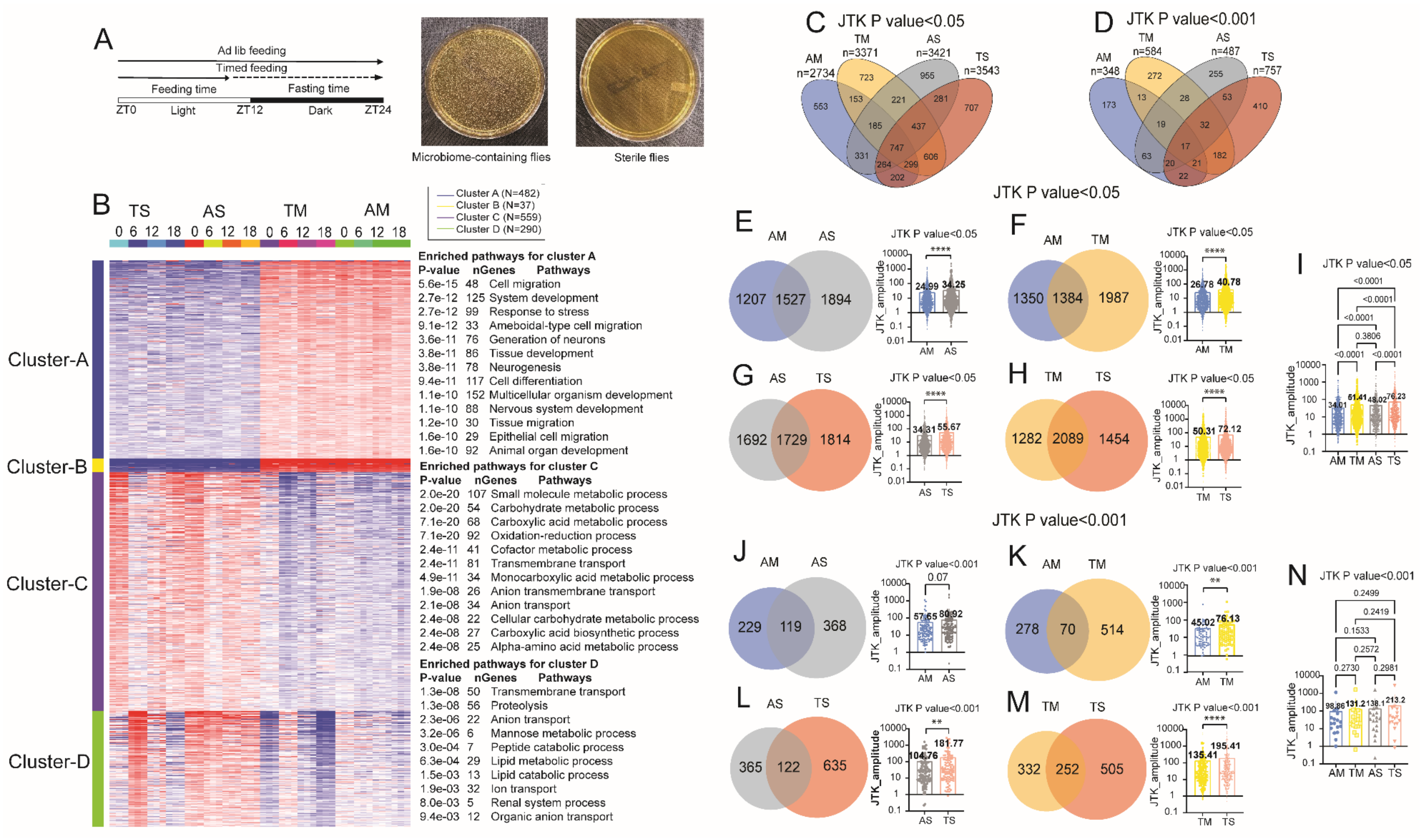
A
Ad lib feeding
Timed feeding
Feeding time
Fasting time
ZT0
Light
ZT12
Dark
ZT24
Microbiome-containing flies
Sterile flies
B
TS AS TM AM
0 6 12 18 0 6 12 18 0 6 12 18 0 6 12 18
Cluster A (N=482)
Cluster B (N=37)
Cluster C (N=559)
Cluster D (N=290)
Cluster-A
Cluster-B
Cluster-C
Cluster-D
Enriched pathways for cluster A
P-value nGenes Pathways
5.6e-15 48 Cell migration
2.7e-12 125 System development
2.7e-12 99 Response to stress
9.1e-12 33 Ameboidal-type cell migration
3.6e-11 76 Generation of neurons
3.8e-11 86 Tissue development
3.8e-11 78 Neurogenesis
9.4e-11 117 Cell differentiation
1.1e-10 152 Multicellular organism development
1.1e-10 88 Nervous system development
1.2e-10 30 Tissue migration
1.6e-10 29 Epithelial cell migration
1.6e-10 92 Animal organ development
Enriched pathways for cluster C
P-value nGenes Pathways
2.0e-20 107 Small molecule metabolic process
2.0e-20 54 Carbohydrate metabolic process
7.1e-20 68 Carboxylic acid metabolic process
7.1e-20 92 Oxidation-reduction process
2.4e-11 41 Cofactor metabolic process
2.4e-11 81 Transmembrane transport
4.9e-11 34 Monocarboxylic acid metabolic process
1.9e-08 26 Anion transmembrane transport
2.1e-08 34 Anion transport
2.4e-08 22 Cellular carbohydrate metabolic process
2.4e-08 27 Carboxylic acid biosynthetic process
2.4e-08 25 Alpha-amino acid metabolic process
Enriched pathways for cluster D
P-value nGenes Pathways
1.3e-08 50 Transmembrane transport
1.3e-08 56 Proteolysis
2.3e-06 22 Anion transport
3.2e-06 6 Mannose metabolic process
3.0e-04 7 Peptide catabolic process
6.3e-04 29 Lipid metabolic process
1.5e-03 13 Lipid catabolic process
1.9e-03 32 Ion transport
8.0e-03 5 Renal system process
9.4e-03 12 Organic anion transport
C JTK P value<0.05
AM n=2734
TM n=3371
AS n=3421
TS n=3543
D JTK P value<0.001
AM n=348
TM n=584
AS n=487
TS n=757
JTK P value<0.05
E AM AS
1207 1527 1894
24.99 34.25
F AM TM
1350 1384 1987
26.78 40.78
G AS TS
1692 1729 1814
34.31 55.67
H TM TS
1282 2089 1454
50.31 72.12
I JTK P value<0.05
34.01 51.41 48.02 76.23
JTK P value<0.001
J AM AS
229 119 368
57.65 80.92
K AM TM
278 70 514
45.02 76.13
L AS TS
365 122 635
104.76 181.77
M TM TS
332 252 505
135.41 195.41
N JTK P value<0.001
98.86 131.2 138.1 213.2
JTK_amplitude

Figure S2. Less and more stringent JTK_P analysis also reveals enhanced transcript cycling in sterile flies and flies subjected to timed feeding

A: Schematic showing experimental protocol for ad lib and timed feeding timepoints in a 12:12 h L:D cycle. Microbiome-containing and sterile flies were separately lysed in sterile PBS buffer and then tested with a *Lactobacilli* MRS agar plate. Only extracts from microbiome-containing flies form colonies. **B:** Heatmap shows the top 1000 differentially expressed transcripts, based on PCA analysis, in fly guts in AS, TS versus AM, TM. Genes with similar expression patterns were identified by K-means clustering and divided into Clusters A-D. Clusters A and B show genes enriched in microbiome-carrying flies relative to sterile flies, and C and D show genes expressed higher in sterile flies than in microbiome-carrying flies. GO biological process enrichment analysis, which is shown on the right. Clusters C and D are composed primarily of metabolic genes**. C:** Venn diagram illustrates the number of overlapping oscillating transcripts between AM, TM, AS and TS. Cycling based on $p < 0.05$, JTK_cycle. **D:** Venn diagram illustrates the number of overlapping oscillating transcripts between AM, TM, AS and TS. Cycling based on $p < 0.001$, JTK_cycle. **E-H:** Venn diagram illustrates the number of overlapping oscillating transcripts and comparison of amplitudes of these transcripts between AM and AS, AM and TM, AS and TS, TM and TS. Cycling based on $p < 0.05$, JTK_cycle. Data are mean ± SEM, ****$p < 0.0001$ shown by Student's t test. **I:** Comparison of amplitudes of cycling transcripts shared across AM, TM, AS and TS. Cycling based on $p < 0.05$, JTK_cycle in a 12:12 LD. Data are mean ± SEM, P value determined by one-way ANOVA and Tukey's multiple comparison test. **J-M:** Venn diagram illustrates the number of overlapping oscillating transcripts and comparison of amplitudes of these transcripts between AM and AS, AM and TM, AS and TS, TM and TS with $p < 0.001$, JTK_cycle. Data are mean ± SEM, ****$p < 0.0001$ shown by Student's t test. **N:** Comparison of amplitudes of cycling transcripts shared across AM, TM, AS and TS. Cycling based on $p < 0.001$, JTK_cycle in a 12:12 LD. Data are mean ± SEM, P value determined by one-way ANOVA and Tukey's multiple comparison test.

Fig.S3

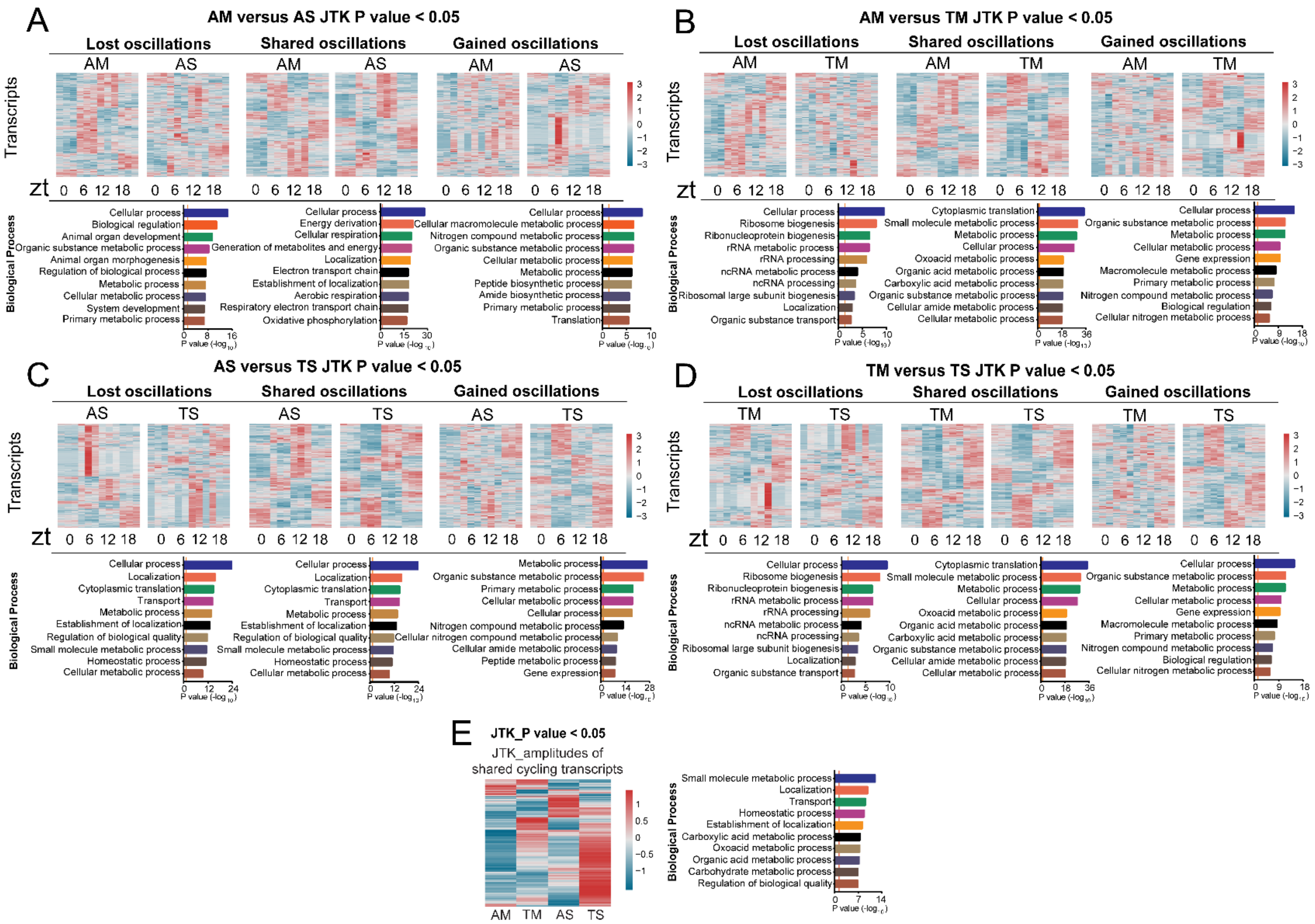
A
AM versus AS JTK P value < 0.05
Lost oscillations
Shared oscillations
Gained oscillations
AM AS AM AS AM AS
Transcripts
zt 0 6 12 18
Biological Process
Cellular process
Biological regulation
Animal organ development
Organic substance metabolic process
Animal organ morphogenesis
Regulation of biological process
Metabolic process
Cellular metabolic process
System development
Primary metabolic process
Energy derivation
Cellular respiration
Generation of metabolites and energy
Localization
Electron transport chain
Establishment of localization
Aerobic respiration
Respiratory electron transport chain
Oxidative phosphorylation
Cellular macromolecule metabolic process
Nitrogen compound metabolic process
Peptide biosynthetic process
Amide biosynthetic process
Translation
P value (-log10)
B
AM versus TM JTK P value < 0.05
AM TM AM TM AM TM
Ribosome biogenesis
Ribonucleoprotein biogenesis
rRNA metabolic process
rRNA processing
ncRNA metabolic process
ncRNA processing
Ribosomal large subunit biogenesis
Organic substance transport
Cytoplasmic translation
Small molecule metabolic process
Oxoacid metabolic process
Organic acid metabolic process
Carboxylic acid metabolic process
Cellular amide metabolic process
Gene expression
Macromolecule metabolic process
Cellular nitrogen compound metabolic process
C
AS versus TS JTK P value < 0.05
AS TS AS TS AS TS
Transport
Regulation of biological quality
Homeostatic process
Cellular nitrogen compound metabolic process
Peptide metabolic process
D
TM versus TS JTK P value < 0.05
TM TS TM TS TM TS
E
JTK_P value < 0.05
JTK_amplitudes of shared cycling transcripts
AM TM AS TS
Small molecule metabolic process
Localization
Transport
Homeostatic process
Establishment of localization
Carboxylic acid metabolic process
Oxoacid metabolic process
Organic acid metabolic process
Carbohydrate metabolic process
Regulation of biological quality
P value (-log10)

Figure S3. Similar functional classes of cycling genes are affected by the presence of a microbiome or by timed feeding, regardless of the stringency of analysis

A: Phase-sorted heatmaps showing transcripts whose oscillations are lost, shared or gained in AS flies versus AM flies. GO biological process enrichment analysis of oscillating transcripts is shown at the bottom. **B:** Phase-sorted heatmaps for transcripts whose oscillations are lost, shared or gained in TM flies versus AM flies. GO biological process enrichment analysis of oscillating transcripts is shown at the bottom. **C:** Phase-sorted heatmaps for transcripts whose oscillations are lost, shared or gained in TS flies versus AS flies. GO biological process enrichment analysis of oscillating transcripts is shown at the bottom. **D:** Phase-sorted heatmaps for transcripts whose oscillations are lost, shared or gained in TS flies versus TM flies. GO biological process enrichment analysis of oscillating transcripts is shown at the bottom. **E:** Heatmap showing the amplitudes of cycling transcripts shared across AM, TM, AS and TS with $p < 0.05$, JTK_cycle in a 12:12 LD. GO biological process enrichment analysis of the shared oscillating transcripts is shown to the right.

Fig.S4

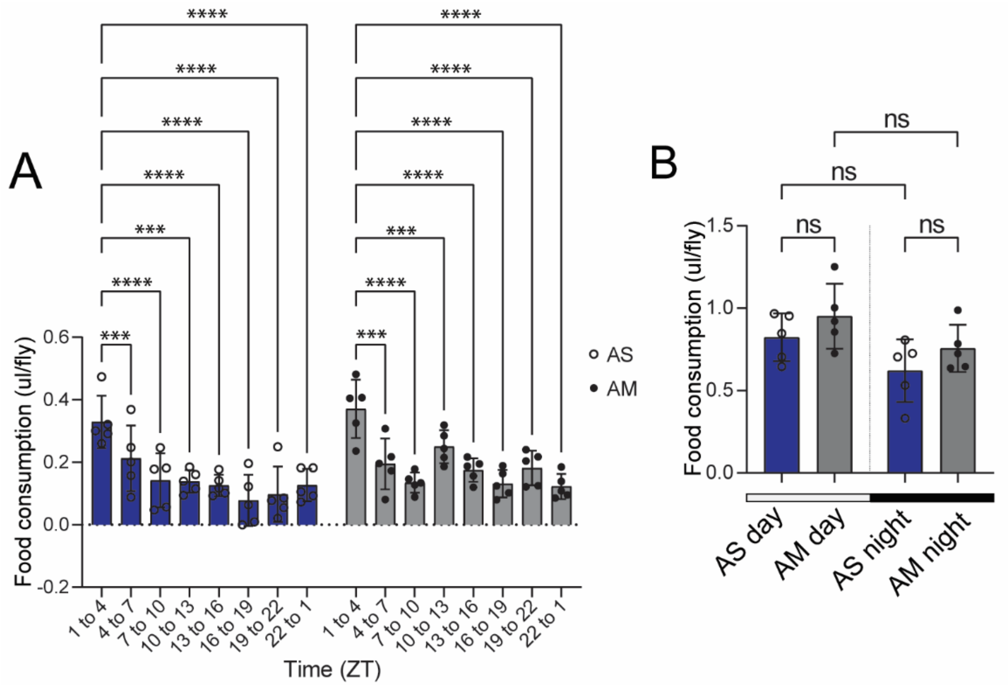

Figure S4. Feeding rhythms do not differ between AS and AM flies
A: Food consumption across 24h in AS and AM male flies. n_{AS}=20, n_{AM}=20, two-way ANOVA with Tukey's multiple comparisons test (*** p adj < 0.001; **** p adj < 0.0001), only relevant comparisons shown. **B:** Total day (average of four measurements from ZT1 to ZT13) and night intake (average of four measurements from ZT13 to ZT1) by AS and AM flies. n_{AS}=20, n_{AM}=20, p<0.05, one-way ANOVA, only relevant comparisons shown.

Fig.S5

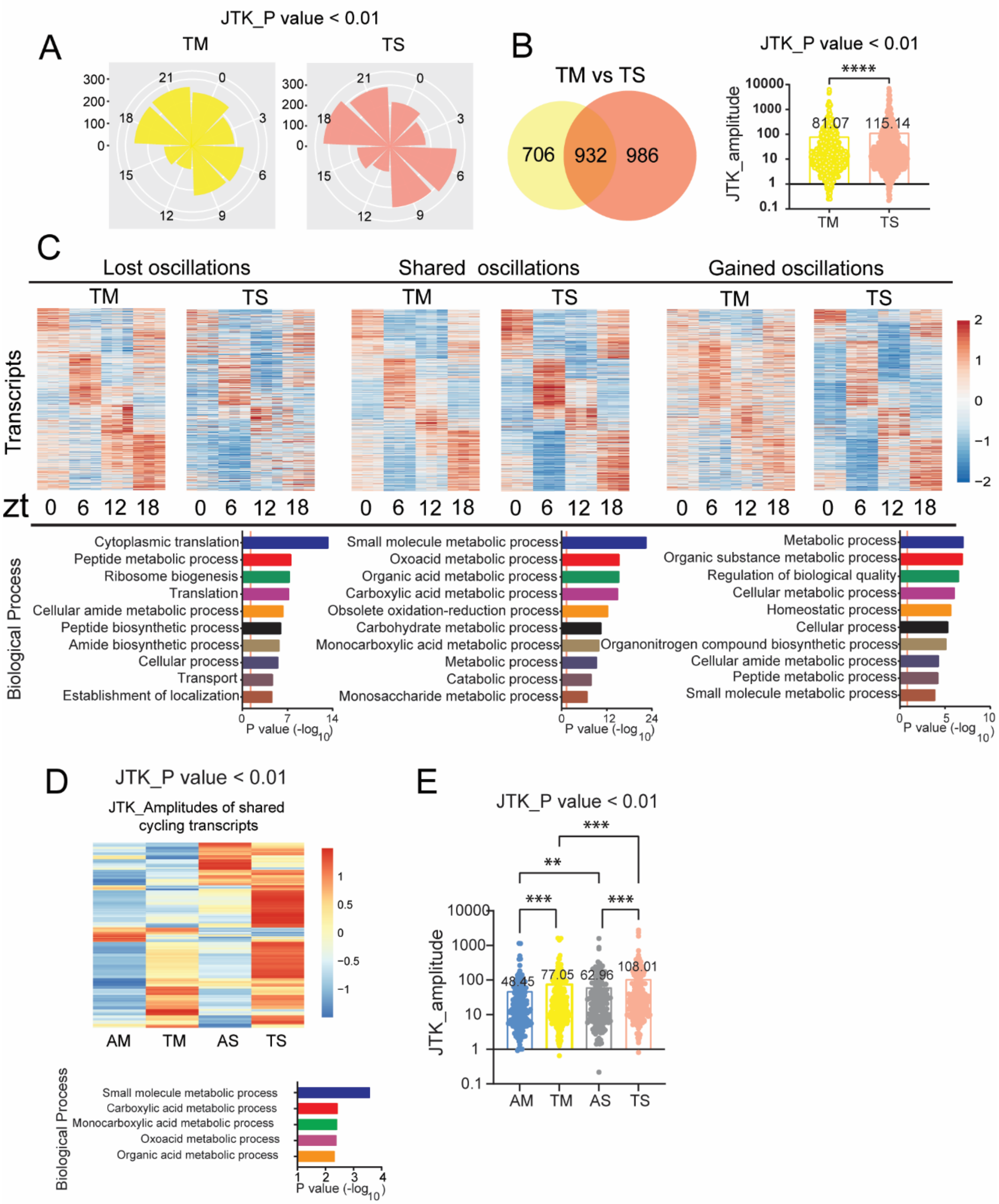

Figure S5. Transcript cycling in timed fed germ-free flies is stronger than in microbiome-containing flies

A: Polar histogram plots of the peak phase for transcripts that cycle in TM and TS conditions. **B:** Amplitude comparison for transcripts that overlap in cycling in TM and TS. Data are mean ± SEM, ****$p < 0.0001$ shown by Student's t test. **C:** Phase-sorted heatmaps for transcripts whose oscillations are lost, shared or gained in TS

flies versus TM flies. GO biological process enrichment analysis of oscillating transcripts is shown at the bottom. **D:** Heatmap showing the amplitudes of cycling transcripts shared across AM, TM, AS and TS with $p < 0.01$, JTK_cycle in 12:12 LD. GO biological process enrichment analysis of the shared oscillating transcripts is shown at the bottom. **E:** Comparison of amplitudes of cycling transcripts shared across AM, TM, AS and TS with $p < 0.01$, JTK_cycle in 12:12 LD. Data are mean ± SEM, **$p < 0.01$ and ***$p < 0.001$ determined by one-way ANOVA and Tukey's multiple comparison test.

Fig.S6

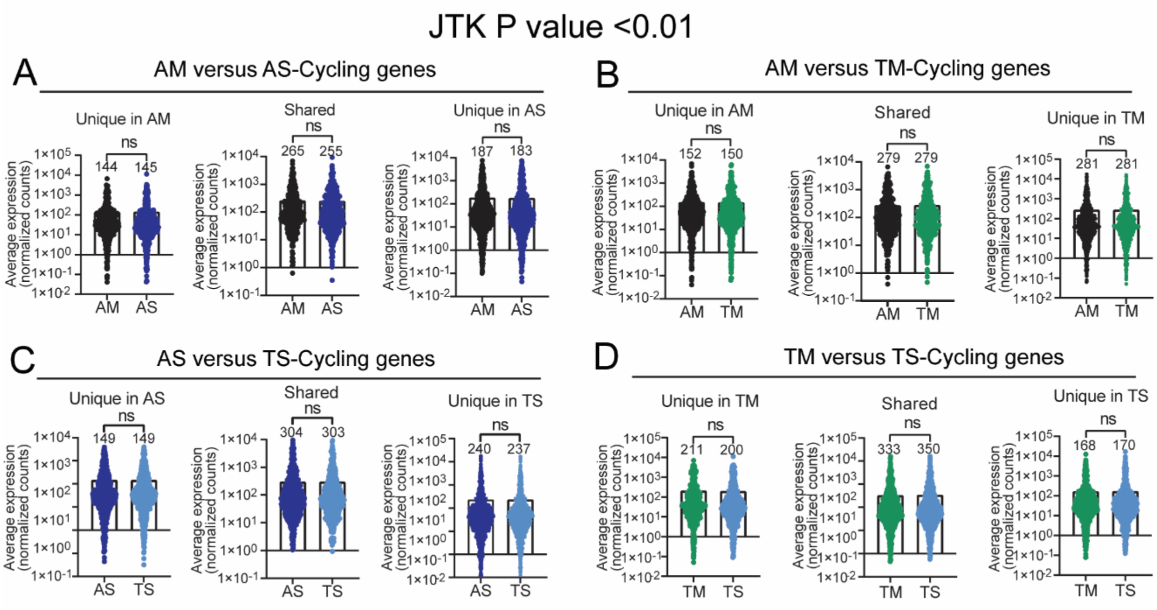

Figure S6. Transcript cycling in germ-free and microbiome-containing flies is independent of changes in average expression levels of the affected genes
A-D: Average expression levels of gut transcripts that cycle in either or both conditions (lost, shared and gained). The number indicates the average normalized counts in each condition for genes that cycle with $p < 0.05$, JTK_cycle. Data are mean ± SEM, comparisons were with Student's t test. A: AS versus AM; B: TM versus AM; C: TS versus AS; D**:** TS versus TM.

Fig.S7

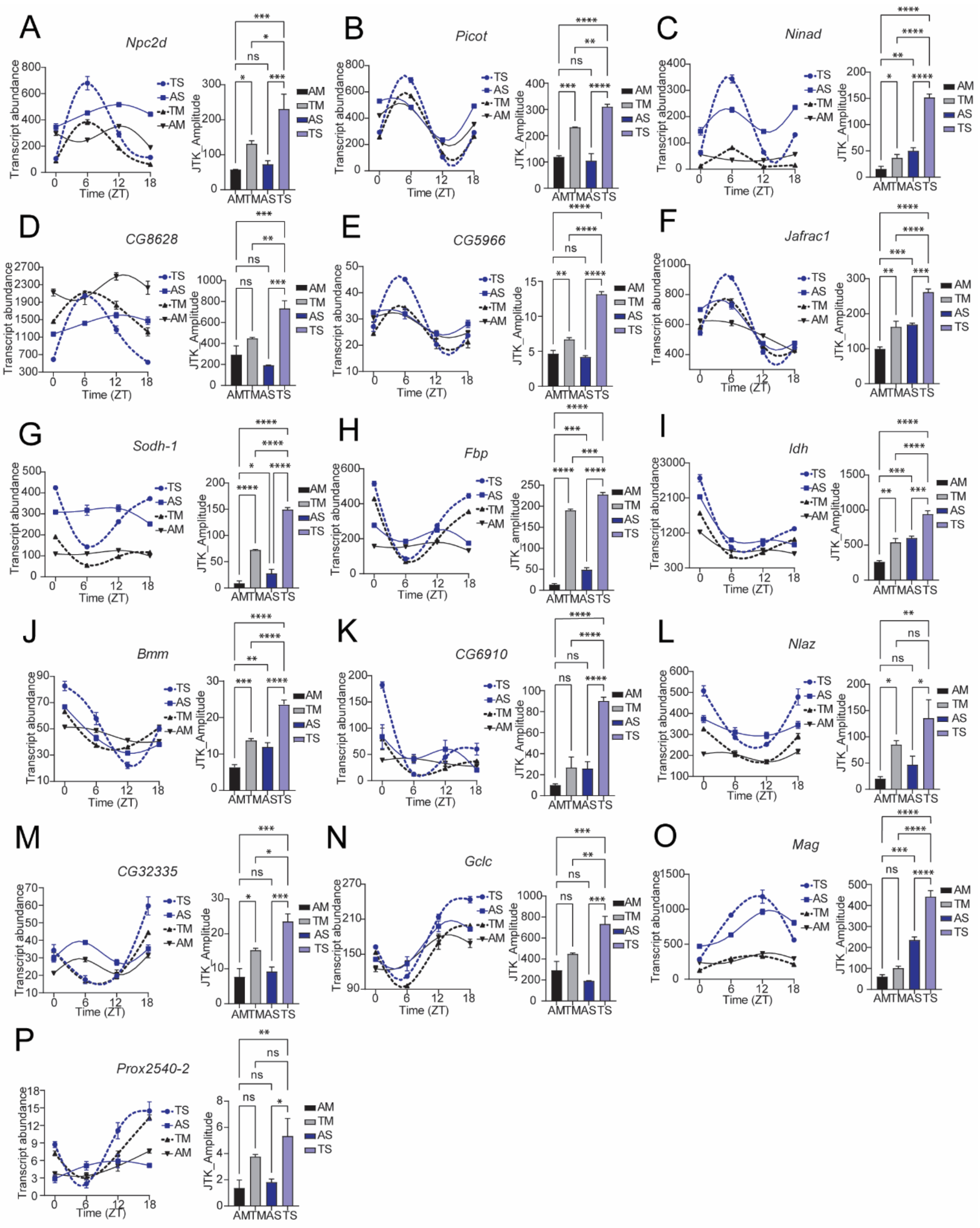

Figure S7. The cycling of metabolic transcripts is modulated by timed feeding and by the microbiome

Transcript abundance and JTK_amplitude analysis of sixteen metabolic genes at different timepoints in a 12:12 L:D cycle under AM, TM, AS and TS conditions (by RNAseq). Data are mean ± SEM, *p < 0.05, **p < 0.01, ***p < 0.001 and ****p <

0.0001 determined by one-way ANOVA and Tukey's multiple comparison test. Based upon the timing of peak expression under TM and TS, as determined by JTK_cycle, transcripts are shown in (**A-F**) peak expression ZT6-9, (**G-L**) peak expression ZT0-3 and (**M-P**) peak expression ZT12-21.

Fig.S8

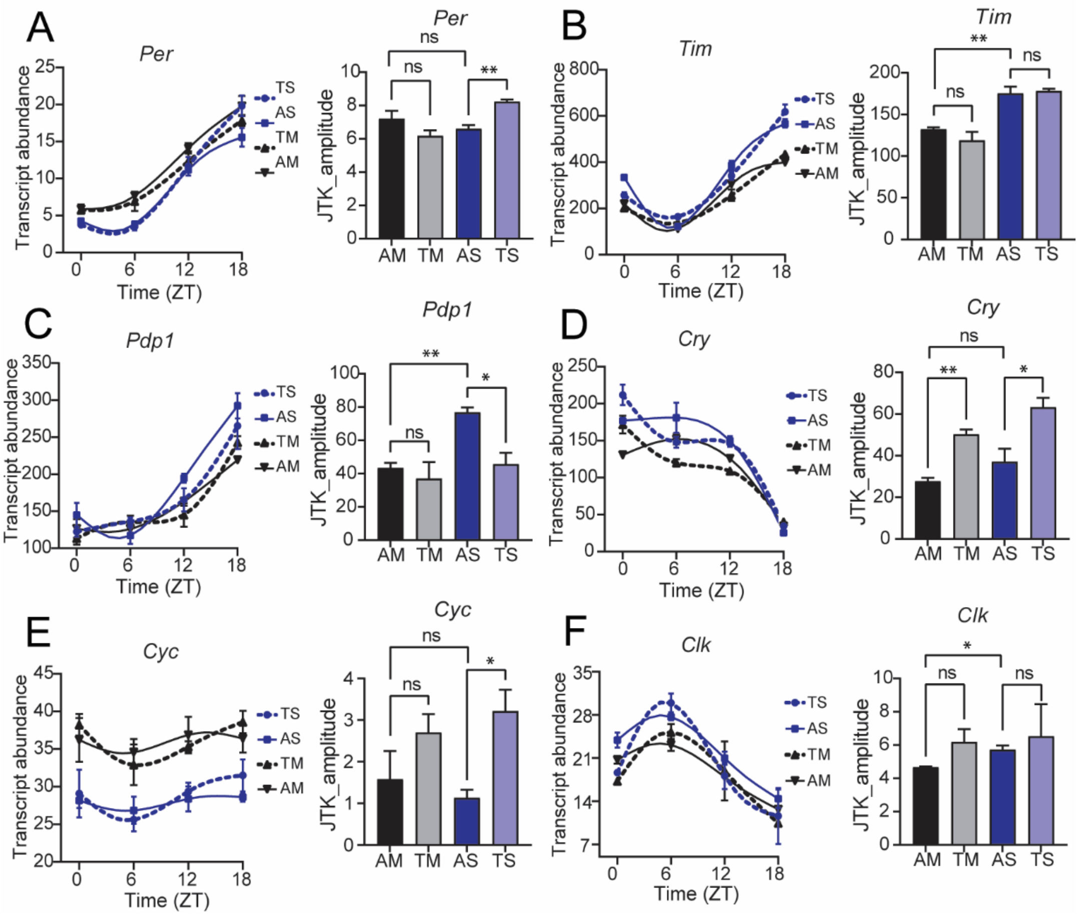

Figure S8. Cycling of clock genes in the gut is affected by loss of the microbiome

A-F: Transcript abundance and JTK_Amplitude analysis of six clock genes at different timepoints in a 12:12 h L:D cycle under AM, TM, AS and TS conditions (by RNAseq). Data are mean ± SEM, **p < 0.05 and *p < 0.05 determined by Student's t test.

Fig.S9

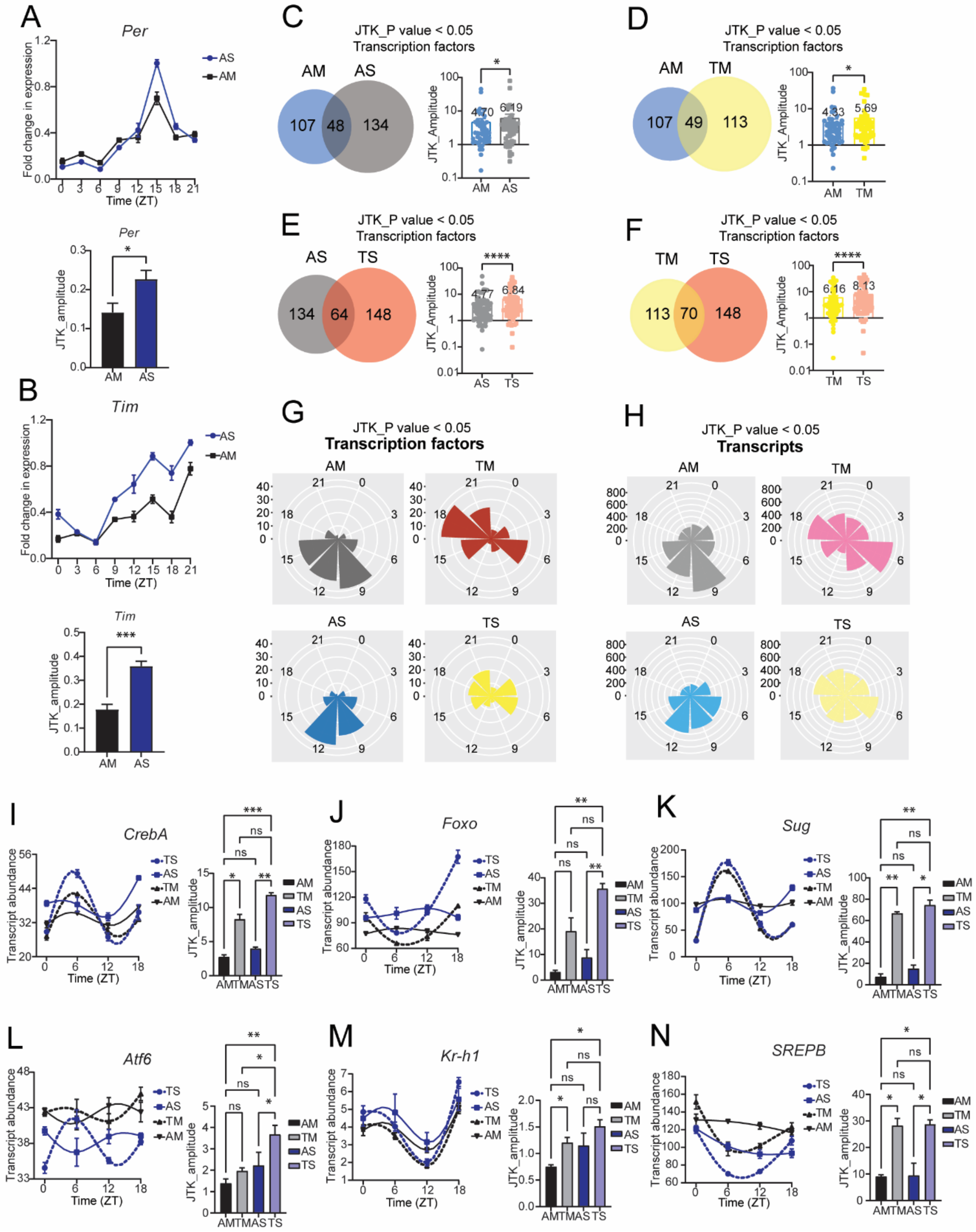

Figure S9. Cycling of clock genes and transcription factors is affected by loss of a microbiome and/or timed feeding

A: Relative expression and JTK_amplitude analysis of *per* cycling in AM and AS guts, assayed via qPCR. Data are mean ± SEM, *p < 0.05 indicated by Student's

t test. N=5 biological replicates. **B**: Relative expression and JTK_amplitude analysis of *tim* cycling in AM and AS guts, assayed via qPCR. Data are shown as mean ± SEM, **$p < 0.01$ indicated by Student's t test. **C-F**: Venn diagrams illustrate the overlap of oscillating transcription factors under the different conditions assayed. (**C:** AM versus AS, **D:** AM versus TM, **E:** AS versus TS and **F:** TM versus TS). Plots on the right show a comparison of amplitudes for cycling transcripts shared by each set of two conditions. $p < 0.05$ by JTK_cycle was used to assess cycling. Data are mean ± SEM, *$p < 0.05$ shown by Student's t test). **G:** Polar histogram plots of the peak phase for oscillating transcription factors under AM, TM, AS and TS conditions, using a JTK_cycle value of $p < 0.05$. The distribution of phases changes with timed feeding, but does not very much in sterile and microbiome-carrying flies. **H:** Polar histogram plots of the peak phase for oscillating transcripts under AM, TM, AS and TS conditions, using a JTK_cycle value of $p < 0.05$. The distribution of phases changes with timed feeding, but does not very much in sterile and microbiome-carrying flies. **I-N:** Transcript abundance and JTK_amplitude analysis of metabolism-related transcription factors at different timepoints in a 12:12 h L:D cycle under AM, TM, AS and TS conditions. Data are mean ± SEM, *$p < 0.05$, **$p < 0.01$, ***$p < 0.001$ and ****$p < 0.0001$ determined by one-way ANOVA and Tukey's multiple comparison test.

Fig.S10

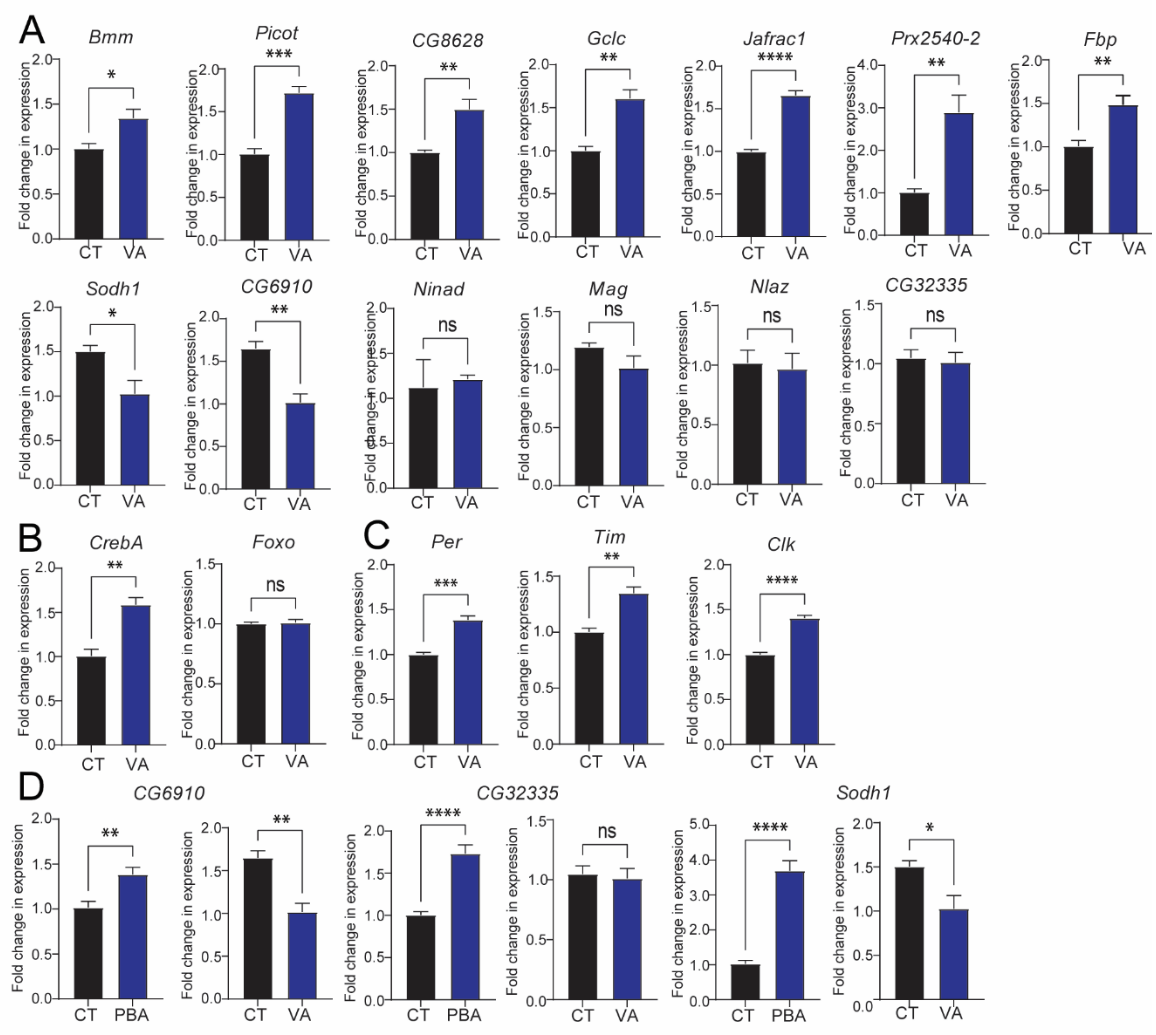

Figure S10. Different HDAC inhibitors may act on different histone acetylation sites

A: Expression of sixteen representative metabolic genes 24-h after injection of VA. CT-Control. Data are mean ± SEM, *p < 0.05, **p < 0.0, ***p < 0.01 and ****p < 0.01 indicated by Student's t test. N=4 biological replicates. **B-C:** Expression of two representative transcription factors (*CrebA* and *Foxo*) and three clock genes (*per*, *tim* and *Clock*) 24-h after injection of VA. Data are mean ± SEM, *p < 0.05, **p < 0.0, ***p < 0.01 and ****p < 0.01 indicated by Student's t test. N=4 biological replicates. **D:** Four metabolic genes show different responses to PBA and VA. Data are mean ± SEM, **p < 0.0, ***p < 0.01 and ****p < 0.01 determined by Student's t test. N=4 biological replicates for VA and 7 for PBA.

Fig.S11

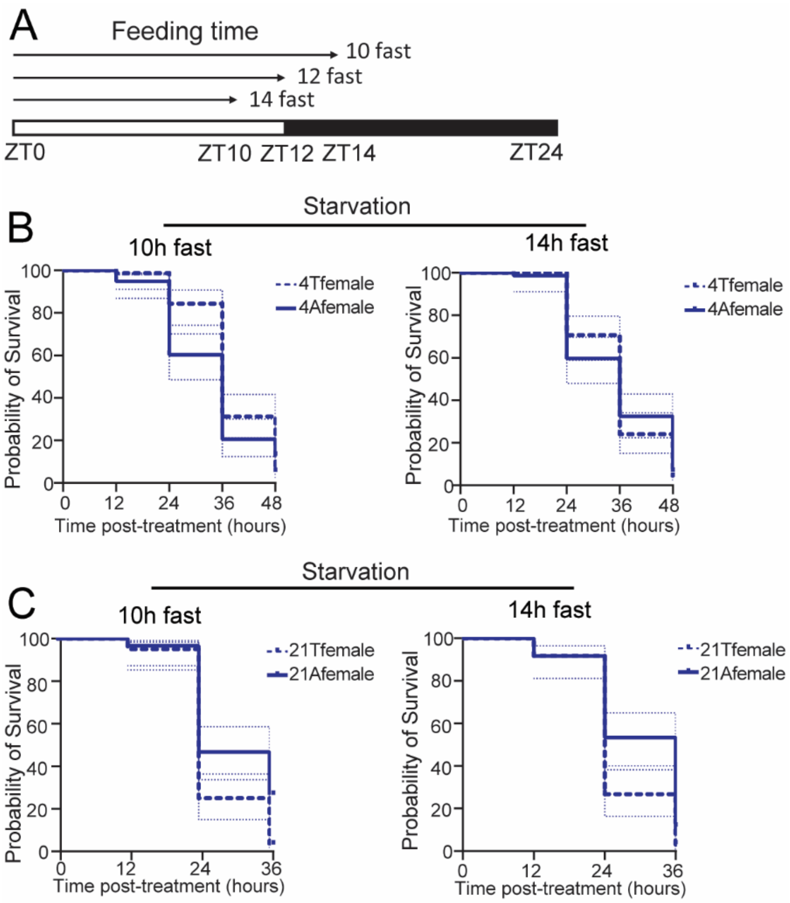

Figure S11. Flies maintained on timed feeding paradigm are more sensitive to starvation

A: Schematic showing experimental protocol for ad lib and timed feeding treatments. Survival curves with 95% confidence interval were generated in response to indicated stressors, N=4-5 biological replicates. **B-C:** Starvation of female flies subjected to ad lib (A) and timed feeding (T) treatments. The timed feeding consisted of a daily overnight 10-h or 14-h fast for 4 days (B) or 21 days (C). For these and all other assays, survival curves were compared via the log-rank (Mantel-cox)–test. Each curve represents four biological replicates of n = 15-16 flies each per condition, Neither the 10-h or 14-h fast for 4 days (B) had a significant effect on response to starvation (p>0.10,). The 21 day TF significantly affected survival for the 10-h (p<0.0006) and 14-h fast (p< 0.0024).

Fig.S12

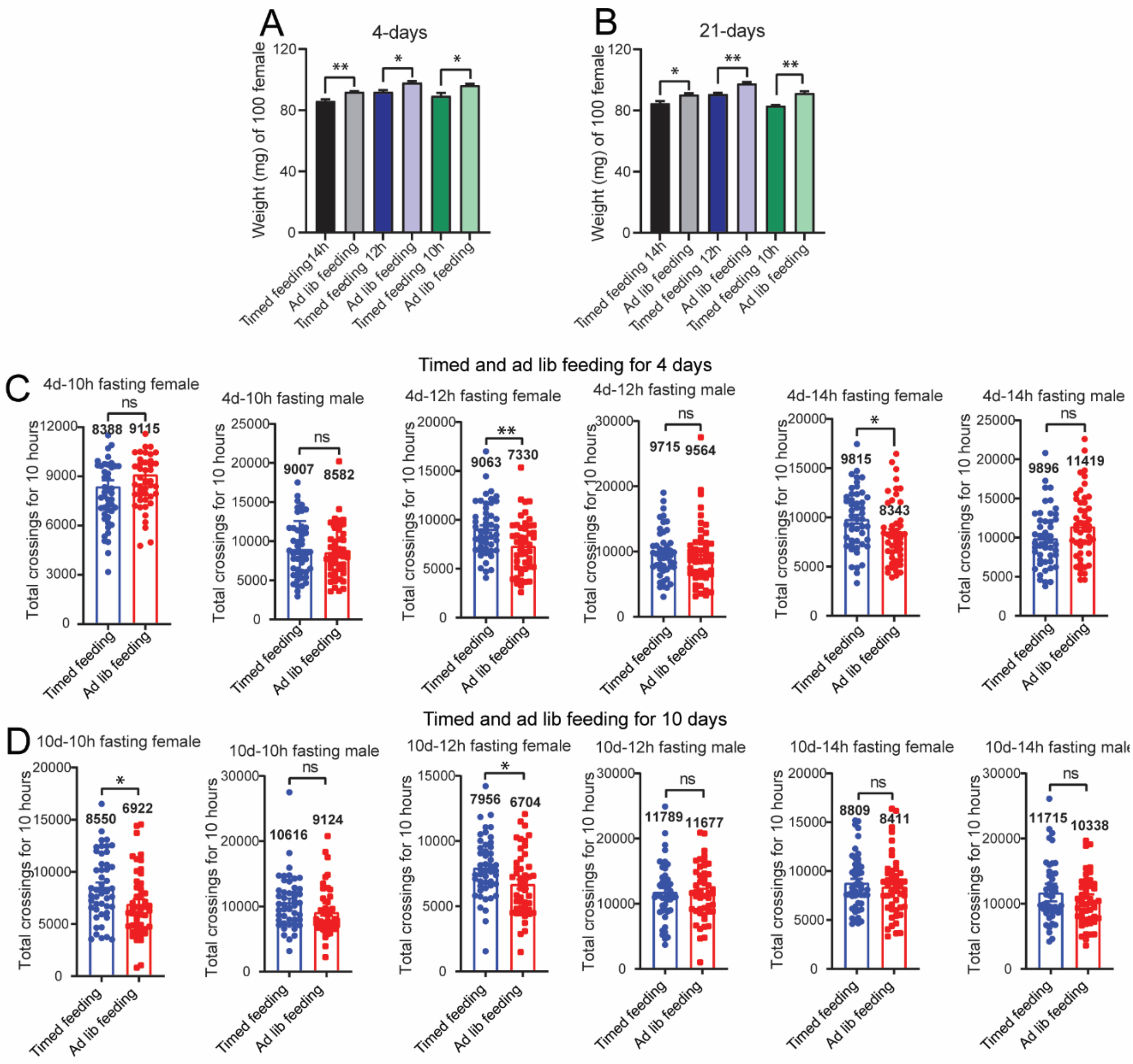

Figure S12. Effect of timed feeding on body weight and locomotor activity (**A** and **B**): Body weight of flies fed ad lib or subjected to timed feeding. Timed feeding consisted of a 10-h, 12-h or 14-h fast each day for 4 (A) or 21 days (B). (C and D): Locomotor activity was measured as beam crossings over a 10 hour period in *Drosophila* using the multibeam Activity Monitoring system (DAMS), n=48 flies in 3 independent replicates. Flies were either ad lib fed or maintained on 10-h, 12-h or 14-h fast each day for 4 (C) or 10 days (D). Data are mean ± SEM, **p < 0.05 and *p < 0.05 determined by Student's t test. Body weight was determined for groups of 100 females.

Fig.S13

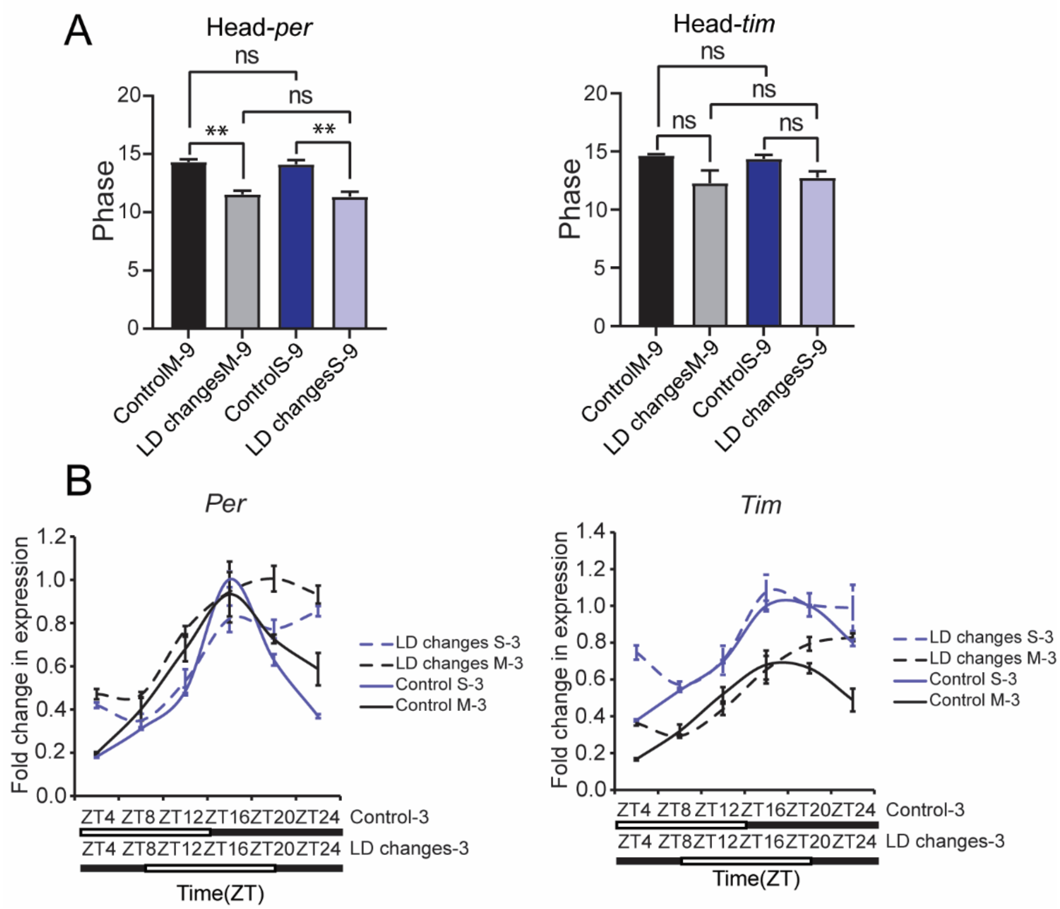

Figure S13. Characterization of *per* and *tim* RNA cycling in heads and guts of sterile and microbiome-carrying flies subjected to shifts of the light:dark cycle

A: Comparison of relative phase of *per* and *tim* RNA in AM (flies with a microbiome) and AS (sterile) heads on the ninth day of LD changes, mRNA levels were measured by qPCR and normalized to the RP49. Data are mean ± SEM (n = 3) *p<0.05, and **p<0.01 determined by Student's t test. Phase is based on meta2d analysis. **B:** Expression pattern of *per* and *tim* in AM and AS fly guts on the third day of LD changes. mRNA levels measured by qPCR are normalized to RP49, data are mean ± SEM (n=4), (Control AM--black line; light:dark changes AM--dashed black line; Control AS--blue line; light:dark changes AS--dashed blue line).

Dataset legends

Dataset **S1A-B:** Gut microbiome sequence data. (A: Diversity measurement analysis with shannon, richness and faith_pd, B: Relative expression abundance of four bacterial species); **S1C-F:** mRNA expression and JTK cycling analysis based on RNA-seq data for AM, TM, AS and TS (C: AM, D: TM, E: AS, F: TS); **S1G:** TM cycling genes in AS; **S1H:** Functional annotation of the 16 metabolic genes; **S1I-L:** Comparison of transcription factors between AM and AS, AM and TM, AS and TS, TM and TS. This includes transcription factors shared by each set of two groups and specific transcription factors for each group (H: AM and AS, I: AM and TM, J: AS and TS, K: TM and TS); **S1M:** The qPCR primers used in this study.

SI References

1. Ja, W. W., Carvalho, G. B., Mak, E. M., de la Rosa, N. N., Fang, A. Y., Liong, J. C., ... & Benzer, S, Prandiology of *Drosophila* and the CAFE assay. *Proc. Natl. Acad. Sci. U.S.A.* 104, 8253-8256 (2007).
